# An inhibitory mono-ubiquitylation of the *Drosophila* initiator caspase Dronc functions in both apoptotic and non-apoptotic pathways

**DOI:** 10.1371/journal.pgen.1006438

**Published:** 2017-02-16

**Authors:** Hatem Elif Kamber Kaya, Mark Ditzel, Pascal Meier, Andreas Bergmann

**Affiliations:** 1 Department of Molecular, Cell and Cancer Biology, University of Massachusetts Medical School, Worcester, Massachusetts, United States of America; 2 Institute for Genetics and Molecular Medicine, Edinburgh Cancer Research Centre, The University of Edinburgh, Edinburgh, United Kingdom; 3 The Breast Cancer Now Toby Robins Research Centre, Institute of Cancer Research, Mary-Jean Mitchell Green Building, Chester Beatty Laboratories, London, United Kingdom; John Hopkins University School of Medicine, Baltimore, UNITED STATES

## Abstract

Apoptosis is an evolutionary conserved cell death mechanism, which requires activation of initiator and effector caspases. The *Drosophila* initiator caspase Dronc, the ortholog of mammalian Caspase-2 and Caspase-9, has an N-terminal CARD domain that recruits Dronc into the apoptosome for activation. In addition to its role in apoptosis, Dronc also has non-apoptotic functions such as compensatory proliferation. One mechanism to control the activation of Dronc is ubiquitylation. However, the mechanistic details of ubiquitylation of Dronc are less clear. For example, monomeric inactive Dronc is subject to non-degradative ubiquitylation in living cells, while ubiquitylation of active apoptosome-bound Dronc triggers its proteolytic degradation in apoptotic cells. Here, we examined the role of non-degradative ubiquitylation of Dronc in living cells *in vivo*, i.e. in the context of a multi-cellular organism. Our *in vivo* data suggest that in living cells Dronc is mono-ubiquitylated on Lys78 (K78) in its CARD domain. This ubiquitylation prevents activation of Dronc in the apoptosome and protects cells from apoptosis. Furthermore, K78 ubiquitylation plays an inhibitory role for non-apoptotic functions of Dronc. We provide evidence that not all of the non-apoptotic functions of Dronc require its catalytic activity. In conclusion, we demonstrate a mechanism whereby Dronc’s apoptotic and non-apoptotic activities can be kept silenced in a non-degradative manner through a single ubiquitylation event in living cells.

## Author Summary

Apoptosis is a programmed cell death mechanism which is conserved from flies to humans. Apoptosis is mediated by proteases, termed caspases that cleave cellular proteins and trigger the death of the cell. Activation of caspases is regulated at various levels such as protein-protein interaction for initiator caspases and ubiquitylation. Caspase 9 in mammals and its *Drosophila* ortholog Dronc carry a protein-protein interaction domain (CARD) in their prodomain which interacts with scaffolding proteins to form the apoptosome, a cell-death platform. Here, we show that Dronc is mono-ubiquitylated at Lysine 78 in its CARD domain. This ubiquitylation interferes with the formation of the apoptosome, causing inhibition of apoptosis. In addition to its apoptotic function, Dronc also participates in events where caspase activity is not required for cell killing, but for regulating other functions, so-called non-apoptotic functions of caspases such as apoptosis-induced proliferation. We found that mono-ubiquitylation of Lysine 78 plays an inhibitory role for these non-apoptotic functions of Dronc. Interestingly, we demonstrate that the catalytic activity of Dronc is not strictly required in these processes. Our *in vivo* study sheds light on how a single mono-ubiquitylation event could inhibit both apoptotic and non-apoptotic functions of a caspase.

## Introduction

In multicellular organisms, cells have a turning point in their lives to commit to either living or dying. Cells which are committed to die can employ different forms of cell death, the most common one being a conserved form of programmed cell death, called apoptosis [1,2]. Apoptosis plays important roles during development, to maintain tissue homeostasis in adult organisms and in response to stress conditions [3,4]. Studies aimed at the elucidation of regulatory pathways of apoptosis are of outstanding importance because dysregulation of apoptosis can lead to many disorders, including neurodegenerative diseases and cancer [5,6]. The fruit fly *Drosophila melanogaster* provides an excellent model system in which to study the molecular mechanisms of apoptosis owing to its genetic conservation with mammals [7], low genetic redundancy of the apoptotic factors, and a variety of well-established genetic techniques that allow to easily manipulate gene function in specific tissue types and even individual cells.

Caspases, a highly conserved family of Cysteine (Cys) proteases, play a pivotal role in the regulation and execution of apoptosis. Caspases are produced as inactive monomeric zymogenes that consist of three domains, an N-terminal pro-domain, a large subunit containing the catalytic Cys residue, and a C-terminal small subunit. There are two types of apoptotic caspases: initiator caspases such as Caspase-2, Caspase-9 and the *Drosophila* ortholog Dronc; and effector caspases such as the Caspase-3, Caspase-7 and the *Drosophila* orthologs Drice and Dcp-1 [8,9]. The prodomains of initiator caspases carry protein/protein interaction motifs such as the Caspase Recruitment Domain (CARD) [10]. The scaffolding protein Apaf-1 and its *Drosophila* ortholog Dark also carry an N-terminal CARD domain [11–14]. In apoptotic cells, through CARD/CARD interactions with Dark, Dronc is recruited into and activated by a death-inducing protein complex, termed apoptosome [15,16]. Effector caspases which have short prodomains without protein/protein interaction motifs, are activated by the apoptosome through proteolytic cleavages between their subunits.

Interestingly, correct stoichiometry between Dronc and Dark molecules is important for execution of apoptosis [17]. There is feedback inhibition between Dronc and Dark. Overexpression of one protein triggers degradation of the other one [17] ensuring that the levels of functional apoptosome units are low under these conditions. Only if both proteins are co-expressed can a significant apoptotic phenotype be recorded.

Inhibitor of Apoptosis Proteins (IAPs) restrict apoptosis by inhibiting caspases [18,19]. IAPs are characterized by the presence of one to three Baculovirus IAP Repeats (BIR) and some bear a C-terminal RING domain that provides E3 ligase activity for ubiquitylation [18,20,21]. In living cells, *Drosophila* IAP1 (Diap1) interacts with Dronc, Drice and Dcp-1 through the BIR domains [22]. Importantly, binding of Diap1 to caspases is not sufficient for their inhibition; ubiquitylation by the RING domain of Diap1 is required for full inhibition of these caspases [22–24]. In dying cells, the pro-apoptotic proteins Reaper (Rpr), Hid and Grim bind to Diap1 and change the E3 ligase activity of the RING domain which promotes auto-ubiquitylation and degradation of Diap1 [25–32]. This leads to release of Dronc from Diap1 inhibition and free Dronc monomers can be recruited into the Dark apoptosome.

Ubiquitylation is a post-translational modification, which results from conjugation of a protein called Ubiquitin to lysine residues of substrates either as a single moiety (mono-ubiquitylation) or by conjugation of ubiquitin chains (poly-ubiquitylation) [33,34]. The fate of a poly-ubiquitylated protein depends on the nature of the ubiquitin linkage. For example, K48 poly-ubiquitylation triggers proteolytic degradation of target proteins, while K63 poly-ubiquitylation regulates non-degradative events such as cell signaling [35–38]. In contrast, mono-ubiquitylation of a protein is usually not associated with protein degradation. Mono-ubiquitylation of target proteins is involved in DNA repair and endocytosis or may regulate translocation and interaction with other proteins [36,37].

Both mammalian and *Drosophila* caspases are subject of regulatory ubiquitylation mediated by IAPs [18,20,21,39–41]. For example, previous studies conducted *in vitro* and by transfection experiments in cell culture demonstrated that in *Drosophila* Dronc is ubiquitylated by Diap1 [23,24,42]. The importance of the RING domain for control of Dronc activity became clear from genetic analysis. *diap1* mutants lacking the RING domain are embryonic lethal due to massive apoptosis [25]. Consistently, loss of the RING domain of Diap1 triggers processing and activation of Dronc [24] suggesting that ubiquitylation negatively regulates Dronc processing and activation. Initially, it was proposed that ubiquitylated Dronc is degraded by the proteasome [42–44]. However, we showed recently that the level of Dronc protein does not increase in proteasome mutants [45] suggesting that Dronc is not subject of proteasome-mediated degradation. In fact, the control of Dronc activity by ubiquitylation is much more complex than initially anticipated. In living cells, free monomeric Dronc is subject to non-degradative ubiquitylation, while processed and activated Dronc in the Dark apoptosome is degraded in a Diap1-dependent manner [17,24]. That raises the question about the nature and function of non-degradative ubiquitylation of free monomeric Dronc in living cells.

Here, we report that in living cells Dronc is mono-ubiquitylated at Lysine 78 (K78) in its CARD domain. To examine the role of K78 mono-ubiquitylation of Dronc, we mutated this residue to non-ubiquitylatable Arginine (K78R). Dronc^K78R^ and Dronc^wt^ display similar enzymatic activities *in vitro*. However, Dronc^K78R^ is easier incorporated into the Dark apoptosome, is more efficiently processed and thus has higher enzymatic activity there. These data suggest that K78 ubiquitylation inhibits incorporation of Dronc into the Dark apoptosome. Surprisingly, *Dronc*^*K78R*^ also suppresses some of the phenotypes associated with catalytic inactivity of Dronc such as lethality, loss of compensatory proliferation and defects in male genitalia rotation. These observations provide evidence that K78 mono-ubiquitylation also controls non-apoptotic functions of Dronc and suggest that not all of the non-apoptotic functions of Dronc require its catalytic activity. In summary, this *in vivo* study provides a mechanistic link of how ubiquitylation of an initiator caspase can control its activity in both apoptotic and non-apoptotic pathways in a non-degradative manner.

## Results

### Dronc is mono-ubiquitylated in living cells

Because available anti-Dronc antibodies perform poorly in immunoprecipitation (IP) experiments, we took advantage of the Gal4/UAS system [46] and expressed Flag-tagged Dronc (Flag-Dronc) [47] ubiquitously using the *daughterless-Gal4* (*da-Gal4*) driver (denoted *da*>*Flag-Dronc*). Expression of *da*>*Flag-Dronc* in whole animals does not cause any significant developmental, apoptotic or lethality phenotypes. To examine the functionality of Flag-Dronc, we tested if it can rescue the lethal phenotype of strong *dronc* mutants (*dronc*^*I24*^/*dronc*^*I29*^) [48]. We indeed observed that *da*>*Flag-Dronc* is able to rescue the pupal lethality caused by *dronc* null mutations and can be activated in the apoptosome (S1 Fig).

**Fig 1:**
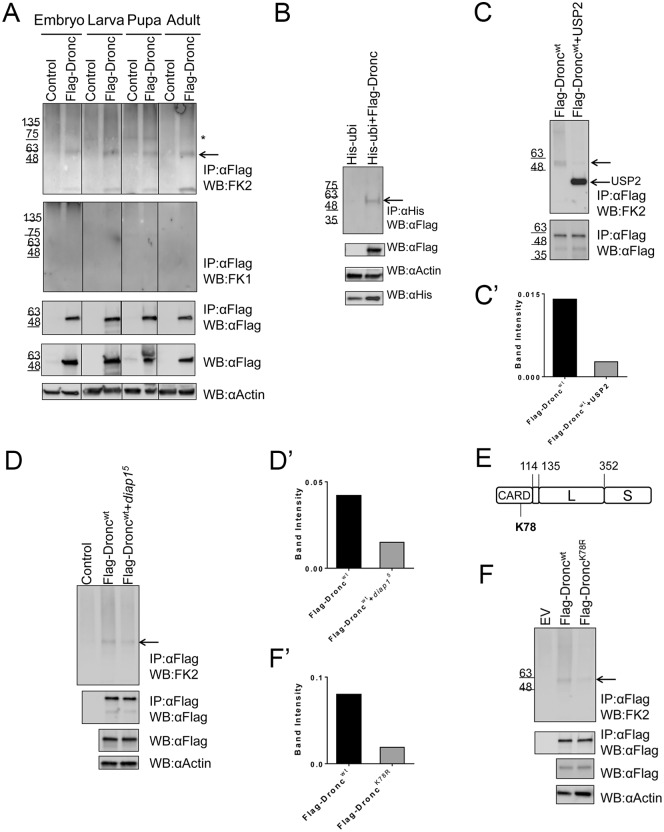
Dronc is mono-ubiquitylated at K78 in living cells for its inhibition. Arrows indicate mono-ubiquitylated Dronc. Asterisks denote unspecific bands. The Flag-Dronc transgene in (A) and (B) is described in [47]. The transgenes used in (C)-(F) and all other figures were generated in this study. **(A)** Immunoprecipitates with Flag antibody from *da*>*Flag*-*Dronc* extracts of the indicated developmental stages were examined for Flag-Dronc ubiquitylation with FK2 and FK1 antibodies. (**B**) Extracts from *da*>*Flag*-*Dronc+6xHis-ubiqitin* larvae were used to pull down 6xHis-tagged ubiquitylated proteins. Flag antibody was used to detect Flag-Dronc. **(C,C’)** USP2 de-ubiquitinase can remove the conjugated mono-ubiquitin on Flag-Dronc. (C’) is the quantification of the Flag-Dronc bands in (C). The FK2 signal in (C’) is normalized against immunoprecipitated Flag-Dronc. The removal of mono-ubiquitin does not cause a significant change in MW of Flag-Dronc. **(D,D’)** Heterozygous *diap1*^*5*^ mutants display reduced Flag-Dronc mono-ubiquitylation (quantified in D’). The loss of mono-ubiquitin does not significantly change the MW of Flag-Dronc. (**E**) Domain structure of Dronc, showing relative position of K78 in the CARD domain. L = large subunit; S = small subunit. (**F,F’**) Flag-Dronc^K78R^ mono-ubiquitylation is significantly reduced compared to Flag-Dronc^wt^. (quantified in F’). The loss of mono-ubiquitin does not significantly change the MW of Flag-Dronc^K78R^.

To address the status of Dronc ubiquitylation, we immunoprecipitated Flag-Dronc from embryonic, larval, pupal and adult fly extracts and blotted with FK1 and FK2 antibodies that bind to ubiquitin-conjugated proteins, but not free, unconjugated ubiquitin. FK2 antibody binds to mono- and poly-ubiquitylated proteins, while FK1 antibody detects only poly-ubiquitin-conjugated proteins [49]. Blotting the IPs with FK2 antibody revealed high molecular poly-ubiquitin species; however, these are comparable to the control IPs and may represent unspecific co-immunoprecipitated proteins (Fig 1A). In contrast, in the 60 kDa range, FK2 detected a single band specifically in Dronc IPs (Fig 1A, arrow). This band is found in all developmental stages tested from embryos to adults. The FK1 antibody did not detect this band (Fig 1A). Flag-Dronc has an estimated molecular weight (MW) of 51 kDa, and adding one ubiquitin moiety of ~8.5 kDa results in a combined MW of about 60 kDa, suggesting that this band may correspond to mono-ubiquitylated Flag-Dronc.

To further verify mono-ubiquitylation of Dronc *in vivo*, we co-expressed *da*>*Flag-Dronc* and 6xHis-tagged ubiquitin (*6xHis-ubiquitin*) and pulled down all ubiquitylated proteins using Ni-NTA agarose beads. Blotting for Flag-Dronc revealed a single band of about 60kDa, that was not present in the control IP in which we only expressed *6xHis-ubiquitin* (Fig 1B). This result further confirms that Dronc is ubiquitylated *in vivo* and the differential detection by FK2, but not FK1, suggests that it is—surprisingly—mono-ubiquitylated.

As further evidence that this modification of Flag-Dronc corresponds to ubiquitylation, we incubated larval Flag-Dronc immunoprecipitates with a de-ubiquitylating enzyme, USP2, that removes conjugated ubiquitin from target proteins. Consistently, in immunoblots, the FK2 signal is strongly reduced after USP2 incubation compared to the control (Fig 1C, upper panel, arrow; quantified in 1C’). Interestingly, although the majority of Flag-Dronc is de-ubiquitylated after USP2 incubation, this does not result in a significant reduction of the molecular weight (MW) of non-ubiquitylated Flag-Dronc (Fig 1C, lower panel). Nevertheless, this characterization indicates that Flag-Dronc is mono-ubiquitylated under *in vivo* conditions.

We were also interested to identify the ubiquitin ligase that mediates mono-ubiquitylation of Dronc. One good candidate is Diap1 which has been shown to ubiquitylate Dronc *in vitro* [23,24,42]. Ideally, to test if Diap1 ubiquitylates Flag-Dronc *in vivo*, one should examine homozygous mutant *diap1* animals for loss of ubiquitylation of Dronc. However, these animals are early embryonic lethal due to strong apoptosis induction by loss of Diap1 [25] which makes this analysis very difficult. Therefore, we examined Flag-Dronc immunoprecipitates from larvae that were heterozygous for the strong *diap1*^5^ allele [26,27]. Immunoprecipitates of Flag-Dronc from heterozygous *diap1*^5^ extracts display a significant reduction of FK2 immunoreactivity (Fig 1D, upper panel; quantified in 1D’) suggesting that Diap1 is involved in mono-ubiquitylation of Flag-Dronc. However, as already noted above in the context of the USP2 experiments, the Flag immunoblots do not display a significant size difference between ubiquitylated and non-ubiquitylated Flag-Dronc (Fig 1D, lower panel). The reason for this unusual behavior is not known.

### Flag-Dronc is ubiquitylated at K78 in the CARD domain

To identify the ubiquitylated Lysine (K) residue, we submitted the 60kDa band from immunoprecipitated Flag-Dronc samples from both larval and pupal stages to mass-spectrometry (LC-MS/MS) analysis. Both analyses showed that Flag-Dronc is ubiquitylated at K78 (S2A Fig). To also examine for poly-ubiquitylation, we submitted higher molecular weight bands of the Flag immunoprecipitates for LC-MS/MS analysis. However, there was no trace of ubiquitylation. In addition to mono-ubiquitylation of K78, we also observed phosphorylation of Ser130, an inhibitory modification of Dronc that has previously been reported [47]. Confirmation of a known modification of Dronc validates the LC-MS/MS approach. Importantly, LC-MS/MS analysis of apoptotic extracts (induced by *hs-hid*) revealed that the mono-ubiquitylation at K78 is absent (S2B Fig). This observation suggests that K78 mono-ubiquitylation is a feature of Dronc in living cells and that it may control (inhibit) the apoptotic activity of Dronc.

To determine whether DIAP1 can ubiquitylate Dronc at K78, we performed *in vitro* ubiquitylation assays of Dronc with Diap1 as E3 ubiquitin ligase and analyzed *in vitro* ubiquitylated Dronc by mass spectrometry. As E2 conjugating enzymes we used either human UBE2D2 or *Drosophila* UBCD1. In both cases, Dronc was found to be ubiquitylated at K78 by DIAP1 *in vitro* (S2C and S2D Fig), suggesting that DIAP1 can mediate K78 ubiquitylation of Dronc.

K78 resides in the CARD domain of Dronc (Fig 1E) which interacts with the CARD domain of Dark for recruitment of Dronc into the apoptosome. To study the role of K78 ubiquitylation, we mutated K78 to Arginine (R) and generated transgenic *UAS*-*Flag-Dronc*^*K78R*^ flies by *phiC31*-based site-specific integration [50,51]. In addition, we combined the K78R mutation with a mutation that changes the catalytic Cys (C) to Ala (A) (C318A), generating transgenic *UAS*-*Flag-Dronc*^*K78RC318A*^ flies. As controls, we generated *UAS*-*Flag-Dronc*^*wt*^, a catalytically inactive Dronc (*UAS*-*Flag-Dronc*^*C318A*^) and empty vector transgenic flies. All constructs are inserted in the same landing site in the genome (VK37 on 2^nd^ chromosome).

To test whether *da*>*Flag-Dronc*^*K78R*^ mutant flies lose the mono-ubiquitylation signal, we immunoprecipitated Dronc from larval samples and probed immunoblots with FK2 antibody. *da*>*Flag-Dronc*^*K78R*^ larval samples showed significantly reduced levels of mono-ubiquitylation (Fig 1F, arrow; quantified in Fig 1F’), suggesting that Flag-Dronc^K78R^ is less efficiently ubiquitylated compared to Flag-Dronc^wt^. However, because K78 is the only Lys residue being detected by LC-MS/MS, we expected a complete loss of ubiquitylation in the Flag-Dronc^K78R^ mutant. Although significantly reduced, the mono-ubiquitylation signal is not completely lost (Fig 1F’) suggesting that in the absence of K78 as major ubiquitin acceptor, another Lys residue may be used as alternative ubiquitylation site (see Discussion). Nevertheless, the K78R mutation revealed that K78 of Dronc is a major ubiquitin acceptor. Interestingly also, as already observed in the USP2 and *diap1*^*5*^ experiments, the MW of ubiquitylated and non-ubiquitylated Dronc is not significantly different (Fig 1F, lower panel).

### Flag-Dronc^K78R^ shows enhanced genetic interaction with Dark in a Diap1-dependent manner

Formation of the apoptosome is essential for activation of Dronc. Interestingly, a recent structural report about the *Drosophila* apoptosome revealed that K78 forms an intramolecular hydrogen bond with a critical residue (Q81) that is required for interaction of the CARD domains of Dronc and Dark for apoptosome formation [16]. Therefore, we hypothesized that mono-ubiquitylation of Dronc at K78 inhibits the interaction with the CARD of Dark, effectively blocking recruitment of Dronc into the apoptosome under surviving conditions. To test this hypothesis *in vivo*, we used genetic and biochemical approaches.

In genetic experiments, we tested whether apoptosis is induced when the K78 mono-ubiquitylation is lost in animals expressing *da*>*Flag-Dronc*^*K78R*^. However, similar to *da*>*Flag-Dronc*^*wt*^, expression of *da*>*Flag-Dronc*^*K78R*^ does not induce a significant apoptotic phenotype or even cause lethality. This is most likely due to the feedback inhibition mechanism between Dronc and Dark according to which overexpressed Dronc destabilizes Dark [17], keeping the number of active apoptosome units low (see Discussion).

**Fig 2:**
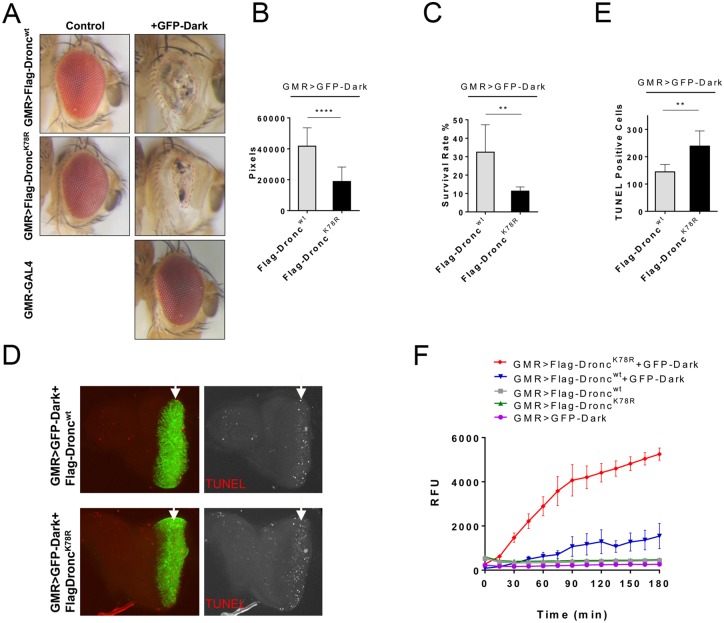
Loss of K78 ubiquitylation results in increased Dronc activity in the apoptosome. (**A-C**) *Flag-Dronc*^*K78R*^ and *GFP-Dark* co-expression under *GMR-Gal4* control results in significantly smaller eyes and a lower survival rate than *GMR*>*Flag-Dronc*^*wt*^*+GFP-Dark*. Control flies just expressing *GMR*>*Flag-Dronc*^*wt*^, *GMR*>*Flag-Dronc*^*K78R*^ or *GMR*>*GFP-Dark* alone, show wild type eye phenotype. (B) Quantification of the eye sizes in (A). n = 19 for *GMR>Flag-Dronc*^*wt*^+*GFP-Dark*; n = 20 for *GMR*>*Flag-Dronc*^*K78R*^+*GFP-Dark*. (C) Quantification of the reduced survival of *GMR*>*Flag-Dronc*^*K78R*^+*GFP-Dark* compared to *GMR*>*Flag-Dronc*^*wt*^+*GFP-Dark*. (**D,E**) Significantly higher TUNEL labeling in the *GMR*-expression domain (arrows) of *GMR*>*Flag-Dronc*^*K78R*^+*GFP-Dark* compared to *GMR*>*Flag-Dronc*^*wt*^+*GFP-Dark* eye imaginal discs of 3^rd^ instar larvae. GFP labels Dark. (E) Quantification of TUNEL positive cells in (D). n = 7 for both genotypes. (**F**) *In vitro* caspase activity assays of adult fly head extracts show significantly higher caspase activity with *GMR*>*Flag-Dronc*^*K78R*^+*GFP-Dark* towards Ac-DEVD-AMC substrate than *GMR*>*Flag-Dronc*^*wt*^+*GFP-Dark*. For quantifications, the student’s t-test was used. Error bars are SD. ** P<0.01; *** P<0,001; **** P<0.0001.

Nevertheless, combined expression of Flag-Dronc^wt^ and Dark (tagged with GFP (GFP-Dark) [17]) with *GMR*-*GAL4* in the posterior eye imaginal disc induces apoptosis, causing eyes of reduced size with pigment loss (Fig 2A) and enhanced pupal lethality. Therefore, we asked whether loss of K78 mono-ubiquitylation causes increased activity of Flag-Dronc^K78R^ in the presence of mis-expressed GFP-Dark [17]. Indeed, we found that the adult eyes of *GMR>Flag-Dronc*^*K78R*^*+GFP-Dark* flies are significantly smaller than *GMR>Flag-Dronc*^*wt*^*+GFP-Dark* eyes (Fig 2A and 2B). In addition, the pupal lethality was significantly increased in *GMR>Flag-Dronc*^*K78R*^*+GFP-Dark* compared to *GMR>Flag-Dronc*^*wt*^*+GFP-Dark* (Fig 2C).

To understand whether this phenotype is due to increased apoptotic activity of Flag-Dronc^K78R^, we examined 3^rd^ instar larval eye discs for apoptosis using TUNEL labeling. Parallel to the adult eye phenotypes, we observed significantly more apoptosis in the *GMR>Flag-Dronc*^*K78R*^*+GFP-Dark* eye imaginal discs (Fig 2D and 2E). In addition, fluorimetric caspase activity assays with extracts from *GMR>Flag-Dronc*^*K78R*^*+GFP-Dark* heads showed a significantly higher cleavage activity towards the synthetic DEVD substrate than *GMR>Flag-Dronc*^*wt*^*+GFP-Dark* (Fig 2F). These data suggest that loss of K78 mono-ubiquitylation increases the apoptotic activity of Dronc^K78R^ in the Dark apoptosome.

To examine the role of Diap1 for K78 mono-ubiquitylation of Flag-Dronc, we compared the eye phenotypes of *GMR>Flag-Dronc*^*wt*^*+GFP-Dark* and *GMR>Flag-Dronc*^*K78R*^*+GFP-Dark* in a heterozygous *diap1*^5^ background. *diap1* heterozygosity strongly enhanced the eye phenotype and lethality of *GMR>Flag-Dronc*^*wt*^*+GFP-Dark* animals (S3 Fig). However, loss of one copy of *diap1* only weakly enhances the eye phenotype and lethality of *GMR>Flag-Dronc*^*K78R*^*+GFP-Dark* animals (S3 Fig). These genetic interaction data suggest that K78 ubiquitylation depends on Diap1.

Dark has a C-terminal caspase cleavage site that is thought to destabilize Dark, thus reducing its apoptosis-promoting activity [17,52]. Consistently, a cleavage resistant version of Dark (Dark^V^) showed a hypermorphic phenotype [52]. Therefore, in theory, Dark^V^ should uncouple the anti-apoptotic feedback of Dronc on Dark. However, experimentally, that was not observed [17]. Co-expression of *GMR*>*Dronc*^*wt*^+*GFP-Dark*^*V*^ caused a similar small eye phenotype compared to *GMR*>*Dronc*^*wt*^+*GFP-Dark*^*wt*^ [17]. Thus, although Dark^V^ was suggested to be more active than Dark^wt^, expression of either transgene with Dronc^wt^ did not change the equilibrium of the apoptosome activation [17]. Therefore, we examined whether co-expression of *Flag-Dronc*^*K78R*^ with *GFP-Dark*^*V*^ under *GMR* control is sufficient to shift the equilibrium of apoptosome formation towards higher induction of apoptosis. Indeed, *GMR>Flag-Dronc*^*K78R*^*+GFP-Dark*^*V*^ executed more apoptosis compared to *GMR>Flag-Dronc*^*wt*^*+GFP-Dark*^*V*^ (S4 Fig). Both the adult eye phenotype and the pupal lethality are worsened significantly in *GMR*>*Dronc*^*K78R*^+*GFP*-*Dark*^*V*^ flies (S4 Fig). These findings are consistent with the notion that Flag-Dronc^K78R^ requires functional Dark for increased activity.

### The K78R mutation increases processing of Dronc through enhanced interaction with Dark

**Fig 3:**
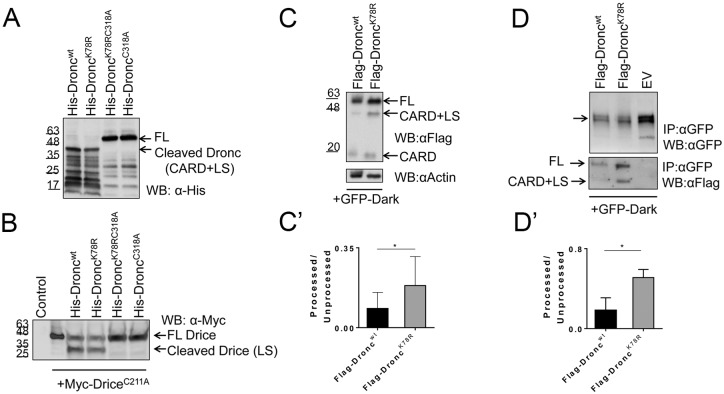
Biochemical characterization of Dronc^K78R^. (**A**) Bacterially expressed 6xHis-Dronc^wt^ and 6xHis-Dronc^K78R^ constructs display similar auto-processing activities. 6xHis-Dronc^C318A^ and 6xHis-Dronc^K78RC318A^ do not show any auto-processing. (**B**) *In vitro* caspase cleavage assays show that 6xHis-Dronc^wt^ and 6xHis-Dronc^K78R^ cleave Myc-Drice^C211A^ with similar activities. 6xHis-Dronc^C318A^ and 6xHis-Dronc^K78RC318A^ cannot cleave Myc-Drice^C211A^. **(C,C’)** 3^rd^ instar lysates of *da*>*GFP-Dark*+*Flag-Dronc*^*wt*^ and *da*>*GFP-Dark*+*Flag-Dronc*^*K78R*^ show that in the presence of Dark, Flag-Dronc^K78R^ is processed significantly more than Flag-Dronc^wt^. In (C’), the average of 4 immunoblots is plotted. (**D**) GFP-Dark interacts with Flag-Dronc^wt^ and Flag-Dronc^K78R^. GFP-immunoprecipitates of 3^rd^ instar larval extracts from *da*>*GFP-Dark*+*Flag-Dronc*^*wt*^, *da*>*GFP-Dark*+*Flag-Dronc*^*K78R*^ and *da*>*GFP-Dark*+*EV* (*Flag-Empty Vector*) animals, probed with anti-GFP antibody (upper panel) and anti-Flag antibody (lower panel). There is a stronger interaction between GFP-Dark and Flag-Dronc^K78R^, resulting in significantly more efficient procession of Flag-Dronc^K78R^ compared to Flag-Dronc^wt^. (**D’**) Relative ratio of processed and unprocessed Flag-Dronc proteins in the Dark apoptosome. Flag-Dronc^K78R^ is more efficiently processed than Flag-Dronc^wt^. The average of 3 immunoblots is plotted. For quantifications, the student’s t-test was used. Error bars are SD. * P<0.05.

To examine if the increased caspase activity of Flag-Dronc^K78R^ is due to increased intrinsic catalytic activity, we performed *in vitro* cleavage assays with bacterially expressed 6xHis-Dronc^wt^, 6xHis-Dronc^K78R^, 6xHis-Dronc^C318A^ and 6xHis-Dronc^K78RC318A^. Because bacteria lack an ubiquitin system, 6xHis-Dronc^wt^ is not modified by ubiquitin enabling us to directly compare the intrinsic activities of the Dronc variants. In these experiments, we first tested the ability of the Dronc constructs to auto-process [53,54]. Both 6xHis-Dronc^wt^ and 6xHis-Dronc^K78R^ proteins are able to auto-process to a similar extend (Fig 3A). In contrast, the catalytic mutant 6xHis-Dronc^C318A^ and double mutant 6xHis-Dronc^K78RC318A^ fail to auto-process (Fig 3A), consistent with the expectation.

Next, we performed *in vitro* cleavage assays of these Dronc preparations with its known cleavage target DrICE [53,54] which is Myc-tagged and carries a mutation in the catalytic site (Myc-Drice^C211A^) to block auto-processing of DrICE. While the catalytic mutants 6xHis-Dronc^C318A^ and 6xHis-Dronc^K78RC318A^ failed to cleave Myc-Drice^C211A^, both 6xHis-Dronc^wt^ and 6xHis-Dronc^K78R^ processed Myc-Drice^C211A^ *in vitro* (Fig 3B). However, the cleavage activities of 6xHis-Dronc^wt^ and 6xHis-Dronc^K78R^ are very similar in these assays suggesting that there are no intrinsic differences in the catalytic activities of 6xHis-Dronc^wt^ and 6xHis-Dronc^K78R^. Furthermore, these data imply that the K78R mutation does not cause any structural defect to Dronc^K78R^. However, *in vivo*, in the presence of Dark, Flag-Dronc^K78R^ has a higher catalytic activity than Flag-Dronc^wt^ (Fig 2) suggesting that Dronc^K78R^ requires Dark for increased catalytic activity.

Consistent with the increased catalytic activity of Flag-Dronc^K78R^ in the presence of Dark, a significantly higher amount of Flag-Dronc^K78R^ is found in the processed form compared to Flag-Dronc^wt^ in immunoblots of total extracts from *da*>*Flag*-*Dronc*^*wt*^ + *GFP*-*Dark* and *da*>*Flag*-*Dronc*^*K78R*^ + *GFP*-*Dark* larvae (Fig 3C and 3C’). To understand the mechanism of increased processing and catalytic activity of Flag-Dronc^K78R^ in the Dark apoptosome, we examined the interaction between Dronc^K78R^ and Dark. Because specific antibodies to Dark do not exist, we used the *GFP*-*Dark* transgenes [17] to immunoprecipitate GFP-Dark and associated Flag-Dronc. To avoid embryonic lethality of *da>Flag-Dronc*^*K78R*^*+GFP-Dark*, *Gal80*^*ts*^ was used to control the expression of *UAS*-*GFP-Dark* and *UAS-Flag-Dronc* transgenes. Using *Gal80*^*ts*^, *da>Flag-Dronc*^*wt*^*+GFP-Dark*, *da>Flag-Dronc*^*K78R*^*+GFP-Dark* and *EV* (empty vector)+*GFP-Dark* as control were induced for 24 h at 29°C and larval extracts were analyzed for Flag-Dronc and GFP-Dark. Longer induction periods (e.g. ≥48 h) also caused lethality. Consistent with a previous report [17], compared to the *EV* control, expression of *da>Flag-Dronc*^*wt*^*+GFP-Dark* and *da*>*Flag*-*Dronc*^*K78R*^+*GFP*-*Dark* reduces Dark’s protein stability, as shown for GFP-Dark in Fig 3D (top panel). In co-IP experiments, we detect an increased interaction between Flag-Dronc^K78R^ and GFP-Dark compared to Flag-Dronc^wt^ and GFP-Dark (Fig 3D, bottom panel). In addition, the ratio between processed versus unprocessed Dronc is significantly increased for Flag-Dronc^K78R^ in complex with GFP-Dark compared to Flag-Dronc^wt^ (Fig 3D, bottom panel; quantified in 3D’), consistent with the increased apoptosis in imaginal discs and head extracts (Fig 2). These results suggest that compared to Flag-Dronc^wt^, Flag-Dronc^K78R^ interacts stronger with Dark and is more efficiently processed for apoptosis induction.

Taken together, these data suggest that living cells are protected from apoptosis by keeping Dronc at least partially inactive through K78 mono-ubiquitylation which appears to block recruitment into the Dark apoptosome. However, when cells are undergoing apoptosis, K78 mono-ubiquitylation is no longer present, allowing Dronc to interact with Dark in the apoptosome and induce cell death.

### K78R is an intragenic suppressor of the lethality associated with loss of catalytic activity of Dronc

**Fig 4:**
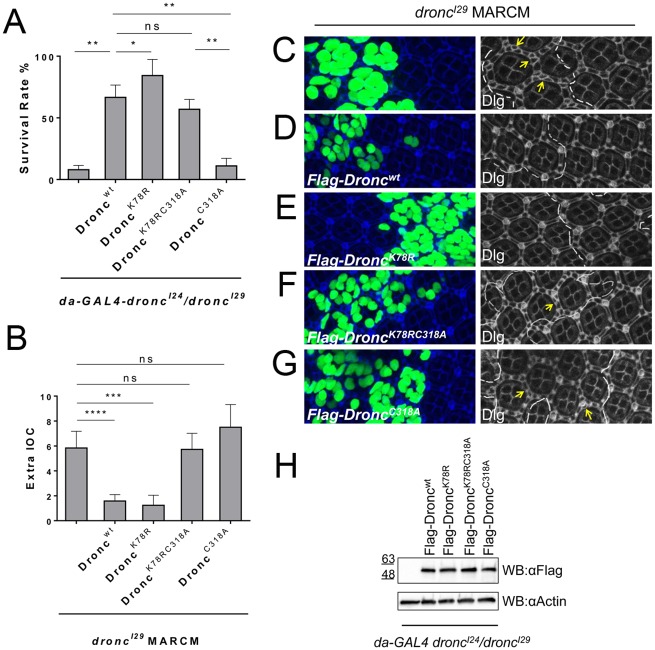
Examination of K78 mono-ubiquitylation with respect to Dronc’s catalytic activity. (**A**) *da>Flag-Dronc*^*wt*^, *da>Flag-Dronc*^*K78R*^ and *da>Flag-Dronc*^*K78RC318A*^ can rescue the lethality of *dronc*^*I29*^ null mutants, whereas *da>Flag-Dronc*^*C318A*^ cannot. (**B**) Quantification of the number of additional interommatidial cells (IOC) shown in (C-G). Genotypes are indicated. MARCM was used to express transgenic *Flag-Dronc* constructs in *dronc*^*I29*^ mutant cell clones. n = 10 for *dronc*^*I29*^ MARCM clones, n = 11 for *Flag-Dronc*^*wt*^ in *dronc*^*I29*^ clones, n = 7 for *Flag-Dronc*^*K78R*^ in *dronc*^*I29*^ clones, n = 11 for *Flag-Dronc*^*K78RC318A*^ in *dronc*^*I29*^ clones, n = 8 for *Flag-Dronc*^*C318A*^ in *dronc*^*I29*^ clones. Each n corresponds to an average of extra IOC of 3 clones. ns—not significant. (**C-G**) Pupal retinae 48h after puparium formation expressing the indicated *Flag-Dronc* constructs in *dronc*^*I29*^ MARCM clones. Clones are marked by GFP and are enclosed by white dashes in the right panels. Examples of extra IOC are marked with yellow arrows. *Flag*-*Dronc*^*wt*^ and *Flag*-*Dronc*^*K78R*^ rescue the IOC phenotype of *dronc* null mutants. However, *Flag*-*Dronc*^*K78RC318A*^ and *Flag*-*Dronc*^*C318A*^ fail to rescue this phenotype. Quantified in (B). **(H)** Immunoblotting of lysates of each Flag-Dronc construct in the *dronc*^*I24*^*/dronc*^*I29*^ background shows similar expression levels. For quantifications, the student’s t-test was used. Error bars are SD. * P<0.05; ** P<0.01; *** P<0,001; **** P<0.0001. ns—not significant.

Next, we examined the physiological role of K78 mono-ubiquitylation of Dronc. For this, we expressed wild-type and mutant *Flag-Dronc* transgenes using *da-Gal4* in a *dronc* null background and scored for rescue. The null mutants used, *dronc*^*I24*^ and *dronc*^*I29*^, have early stop codons at positions 28 and 53, respectively [48] and do not produce any Dronc protein. *dronc*^*I24*^*/dronc*^*I29*^ null mutants display a strong semi-lethal phenotype. Less than 10% of the expected *dronc* homozygous mutant animals survive development (Fig 4A) and hatch as adults with wing abnormalities (S5 Fig) [48]. Expression of *da*>*Flag*-*Dronc*^*wt*^ rescues the lethality of *dronc* null mutant flies, but it is only a partial rescue. There is still about a 35% lethality (Fig 4A), suggesting that *da*>*Flag*-*Dronc*^*wt*^ does not reach sufficient Dronc activity for full rescue. Interestingly, however, *da*>*Flag*-*Dronc*^*K78R*^ rescued the lethality of *dronc* null mutant significantly better than *da*>*Flag*-*Dronc*^*wt*^. More than 80% of the expected progeny emerges as adults in the presence of Flag-Dronc^K78R^ (Fig 4A). Because these transgenes were obtained by phiC31 integration in the same landing site, the expression levels of all Flag-Dronc constructs are comparable (Fig 4H) and are not responsible for the observed differences. Therefore, this result further supports the notion that Flag-Dronc^K78R^ has more activity than Flag-Dronc^wt^ and thus can better substitute for the loss of endogenous *dronc*.

As expected, expression of catalytically inactive *da*>*Flag-Dronc*^*C318A*^ failed to rescue the lethality of *dronc* null mutants (Fig 4A). Surprisingly, however, expression of *da*>*Flag-Dronc*^*K78RC318A*^ which lacks the K78 mono-ubiquitylation site and is catalytically inactive (Fig 3A and 3B), did rescue the lethality of *dronc* null mutants to a significant degree! About 60% of *dronc* mutant flies survived when expressing *da>Flag-Dronc*^*K78RC318A*^ compared to only 10% of *dronc* mutant flies expressing *da*>*Flag-Dronc*^*C318A*^ (Fig 4A). Thus, the K78R mutation behaves as an intragenic suppressor of the lethality associated with loss of catalytic activity of Dronc. This result suggests that loss of K78 ubiquitylation can be advantageous for the survival of *dronc* mutant flies and can even—at least partially- overcome loss of the catalytic activity of Dronc.

### Flag-Dronc^K78RC318A^ does not rescue the apoptotic phenotype of *dronc* null mutants

Because of the intragenic suppression of the lethality of the catalytic *dronc*^*C318A*^ mutant by the K78R mutation, we considered—although did not expect—that the K78R mutation would rescue the catalytic activity of Dronc^C318A^ and thus the apoptotic phenotype of *dronc* mutants. To test this possibility, we employed the developing *Drosophila* retina which consists of individual units called ommatidia. In developing *Drosophila* retinae, cells produced in excess between ommatidia (interommatidial cells, IOCs) are eliminated by apoptosis around 28-30h after puparium formation (APF) [55–58]. The retinal lattice is fully differentiated at 42-45h APF. Previous studies showed that *dronc*^*I24*^ and *dronc* ^*I29*^ mutants fail to remove excess IOCs during development; about six additional IOCs remain per ommatidium in *dronc* mutants (Fig 4B and 4C) [48,59]. To understand the relationship between K78 mono-ubiquitylation and catalytic inactivity during developmental apoptosis, we generated *dronc*^*I29*^ mutant clones expressing *Flag-Dronc*^*wt*^, *Flag-Dronc*^*K78R*^, *Flag*-*Dronc*^*C318A*^ and *Flag-Dronc*^*K78RC318A*^ by MARCM and examined the ability of these constructs to restore IOC apoptosis in the pupal retina of *dronc* mosaics. As expected, while expression of *Flag-Dronc*^*wt*^ and *Flag-Dronc*^*K78R*^ rescues IOC apoptosis in *dronc*^*I29*^ mutant clones, *Flag-Dronc*^*C318A*^ does not (Fig 4D, 4E and 4G; quantified in Fig 4B). Importantly, although expression of *Flag-Dronc*^*K78RC318A*^ rescued the lethality of *dronc* mutant flies (Fig 4A), it does not restore IOC apoptosis in *dronc* mutant clones (Fig 4B and 4F). Consistently, *da*>*Flag-Dronc*^*K78RC318A*^ expression in *dronc* null background does not rescue the wing phenotype of *dronc* mutants (S5E Fig). In addition, Flag-Dronc^K78RC318A^ does not have catalytic activity *in vitro* (Fig 3A and 3B).

Therefore, as expected, these findings suggest that the K78R mutation does not restore the catalytic activity of *Flag-Dronc*^*K78RC318A*^. They further suggest that the suppression of the pupal lethality of *dronc* mutants by expression of *Flag-Dronc*^*K78RC318A*^ occurs independently of the catalytic activity of Dronc which is therefore not absolutely essential for the survival of the flies. These data further imply that K78 mono-ubiquitylation controls additional, non-catalytic (apoptosis- and effector-caspase-independent) functions of Dronc whose failure in *dronc* mutants contributes to lethality.

### K78 ubiquitylation of Dronc is involved in control of apoptosis-induced proliferation

**Fig 5:**
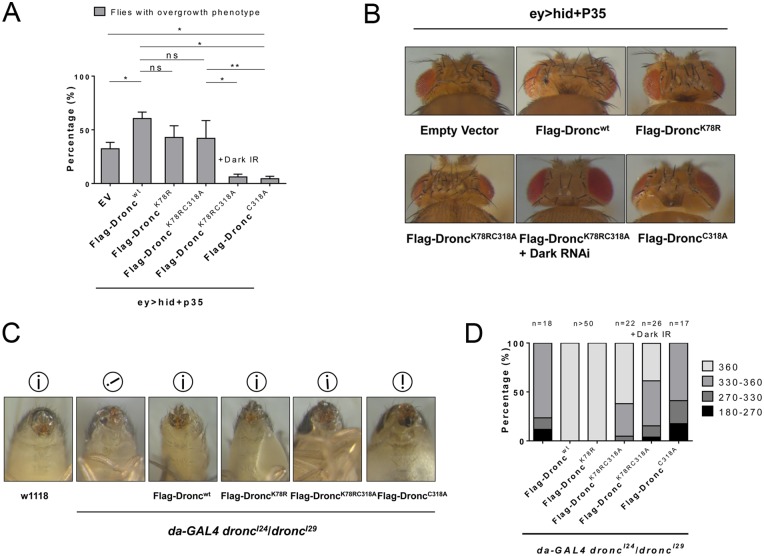
K78 ubiquitylation plays inhibitory roles for additional functions of Dronc. (**A**) Quantification of the enhanced head overgrowth phenotype of *ey>hid+p35* animals expressing the indicated *Flag-Dronc* transgenes. Overgrowth is characterized by expanded head cuticle with pattern duplications such as bristles and ocelli (see examples in (B)). *Flag-Dronc*^*C318A*^ acts in a dominant negative manner in *ey>hid+p35* background. Flag-Dronc^K78RC318A^ phenotype in *ey*>*hid*+*p35* background is dependent on Dark as observed by 94% suppression of the overgrowth phenotype when *dark* RNAi is expressed. **(B)** Representative head phenotypes of *ey>hid+p35* animals expressing the indicated *Flag*-*Dronc* transgenes. **(C)** Male genitalia rotation defect of *dronc* null mutants is fully suppressed by *da*>*Flag-Dronc*^*wt*^ and *da*>*Flag-Dronc*^*K78R*^ (100% of males display 360° rotation) (quantified in D) and partially suppressed by *da*>*Flag-Dronc*^*K78RC318A*^ (62% of males display 360° rotation). *da*>*Flag*-*Dronc*^*C318A*^ failed to suppress this phenotype. The i surrounded by a circle indicates the relative orientation of the male genitalia in the depicted animals (i = wild-type). The suppression by *da*>*Flag-Dronc*^*K78RC318A*^ is partially reverted (38% of full rotation) when Dark RNAi is expressed (quantified in B). **(D)** Quantification of male genitalia rotation defect phenotype in *dronc* null mutants, expressing the indicated *Flag-Dronc* transgenes. For quantifications, the student’s t-test was used. Error bars are SD. * P<0.05; ** P<0.01; ns—not significant.

Next, we examined whether K78 mono-ubiquitylation is involved in a non-apoptotic function of Dronc. We and others have shown that Dronc can trigger apoptosis-induced proliferation (AiP) of neighboring surviving cells independently of downstream effector caspases and thus apoptosis [44,60–62]. Expression of the effector caspase inhibitor P35 is used to uncouple AiP from apoptosis. This treatment blocks apoptosis, but triggers chronic Dronc activity which causes tissue overgrowth due to permanent AiP [44,61–65]. It was previously shown that co-expression of *p35* with *dronc* or pro-apoptotic *hid* using *ey*-*Gal4* (*ey*>*dronc*+*p35* or *ey*>*hid*+*p35*) in eye imaginal discs causes head overgrowth with pattern duplications, while expression of catalytically inactive *ey*>*dronc*^*C318S*^+*p35* did not [61,62,65]. Consistently, expression of *Flag-Dronc*^*wt*^ and *Flag-Dronc*^*K78R*^ in *ey*>*p35* or *ey*>*hid*+*p35* background induced or enhanced head overgrowth, respectively, while catalytically inactive *Flag-Dronc*^*C318A*^ displayed wild-type head phenotypes in these assays (Fig 5A and 5B; S6A Fig). Surprisingly, however, expression of *Flag-Dronc*^*K78RC318A*^ in *ey*>*hid*+*p35* and *ey*>*p35* assays also showed a similar overgrowth phenotype compared to *Flag-Dronc*^*wt*^ or *Flag-Dronc*^*K78R*^ (Fig 5A and 5B; S6A Fig). Thus, similar to the results obtained in the rescue crosses of *dronc* induced lethality, loss of K78 ubiquitylation can suppress loss of catalytic activity in AiP. As controls, we expressed *Flag*-*Dronc* constructs with *ey*-*GAL4* in the absence of *p35*. However, simple overexpression of the *Flag-Dronc* construct did not trigger any overgrowth phenotype in these crosses (S6B Fig).

Because we showed in Figs 2 and 3, that Flag-Dronc^K78R^ interacts better with GFP-Dark than Flag-Dronc^wt^, we wondered if the rescue of AiP by Flag-Dronc^K78RC318A^ is dependent on the interaction with Dark. Indeed, in the absence of Dark (by RNAi), Flag-Dronc^K78RC318A^ is no longer able to restore AiP in *ey*>*hid*+*p35* background (Fig 5A and 5B).

### K78 ubiquitylation of Dronc is involved in control of male genitalia rotation

During development, *Drosophila* male genitalia make a full 360° clockwise rotation [66]. When components of the apoptotic machinery (*hid*, *dronc*, *drICE*) are impaired, the rotation fails or is incomplete [67–70] suggesting that it is an apoptosis-driven event. We examined whether expression of *da*>*Flag-Dronc* constructs could rescue the genitalia rotation defect in *dronc*^*I24*^/*dronc*^*I29*^ males. *da*>*Flag-Dronc*^*wt*^ and *da*>*Flag-Dronc*^*K78R*^ fully rescued the male genitalia rotation phenotype of *dronc* mutant males (100% of males display 360° rotation) (Fig 5C; quantified in Fig 5D). In addition, these males were fertile. In contrast, *da*>*Flag-Dronc*^*C318A*^ was unable to rescue the *dronc*^*I24*^/*dronc*^*I29*^ rotation defect and had incomplete rotations ranging from 180° to 270° (Fig 5C and 5D). These males were also sterile. Interestingly, *da*>*Flag-Dronc*^*K78RC318A*^ partially rescued the rotation defect associated with *dronc* null mutations (62% of males display 360° rotation) (Fig 5C and 5D). However, sterility caused by *dronc* null mutations was not suppressed suggesting that other non-apoptotic processes such as sperm maturation are not rescued [71]. The partial rescue of the rotation phenotype by Flag-*Dronc*^*K78RC318A*^ is potentially interesting because it may suggest that Dronc has two functions for male genitalia rotation: in addition to the previously reported effector caspase-dependent function [69,70], it may also have an effector caspase-independent function. Because effector caspases require catalytic activity of Dronc for activation, only the effector caspase-independent function can be rescued by Flag-*Dronc*^*K78RC318A*^, giving rise to the observed partial rescue (Fig 5C and 5D). The rescue of the rotation phenotype by Flag-*Dronc*^*K78RC318A*^ is also dependent on Dark—at least partially—as *dark* RNAi reduces the rescue to 38% full rotation (Fig 5D). These data further suggest that K78R mutation is an intrinsic suppressor of loss of Dronc’s catalytic activity.

## Discussion

### Implications of K78 mono-ubiquitylation for apoptotic functions of Dronc

Our *in vivo* data uncovered an elegant mechanism of how Dronc activation is regulated through mono-ubiquitylation and how this modification affects both catalytic and non-catalytic functions of Dronc. Our MS/LC-MS data from larval and pupal samples demonstrate that in living cells, Dronc is mono-ubiquitylated at K78. Because mono-ubiquitylation is not a mark for proteasome-mediated degradation, this finding explains why monomeric Dronc is not degraded in living cells [24]. Mono-ubiquitylation of Dronc is not an unprecedented observation in the caspase field. It was previously reported that cIAP2 promotes mono-ubiquitylation of the effector caspases Caspase-3 and Caspase-7 *in vitro* [72]. However, the significance of this mono-ubiquitylation is not known. Furthermore, the paracaspase MALT1 is subject to mono-ubiquitylation [73,74]. Interestingly, this modification leads to MALT1 activation. Here, we add the initiator caspase Dronc in *Drosophila* to the list of caspases being mono-ubiquitylated.

Mono-ubiquitylation of K78 of Dronc does not regulate the intrinsic catalytic activity of Dronc. Purified recombinant Dronc^wt^ and Dronc^K78R^ have comparable catalytic activities *in vitro*. However, the location of K78 in the CARD domain suggests a regulatory modification for the interaction with Dark. Consistently, K78 was recently reported to be a critical residue for the interaction between the CARD domains of Dronc and Dark [16]. Indeed, our genetic analysis suggests that Dronc^K78R^ increases the physical association with Dark, resulting in increased processing of Dronc and thus higher apoptotic activity. Thus, we propose that in living cells, K78 mono-ubiquitylation of Dronc prevents the interaction with Dark.

Because of the increased processing and activation of Dronc^K78R^, we expected a very strong apoptotic phenotype when expressing *Dronc*^*K78R*^ in flies. However, although we observed increased apoptosis by expression of *Dronc*^*K78R*^ compared to *Dronc*^*wt*^, it was not as severe as expected and depended on the presence of mis-expressed Dark. There are a few possibilities to explain this result. Although K78 was identified as the only ubiquitin acceptor site by LC-MS/MS analyses, we did not see a complete loss of mono-ubiquitylation in *Flag-Dronc*^*K78R*^ flies. It is possible that when this major ubiquitin acceptor site is mutated, another Lys residue is selected for ubiquitylation. Nevertheless, the partial loss of ubiquitylation in Dronc^K78R^ (Fig 1E) is sufficient to shift Dronc activity to a higher level. This increased activity depends on the presence of Dark.

Another possibility to explain the absence of a significant apoptotic phenotype of *da*>*Flag*-*Dronc*^*K78R*^ is that correct stoichiometry between Dronc and Dark molecules is important for execution of apoptosis [17]. These proteins mutually control their stability. Overexpression of one protein triggers degradation of the other one [17]. This balance ensures that the levels of functional apoptosome units are low and this is most likely the reason why expression of each protein by itself in a tissue or even in the whole animal does not cause a significant apoptotic phenotype or complete lethality [17]. Only if both proteins are co-expressed can a significant apoptotic phenotype be recorded and under those conditions can Dronc^K78R^ trigger a stronger apoptotic phenotype compared to Dronc^wt^, as observed in Fig 2. Nevertheless, it should be pointed out that there are also conditions under which mis-expression of Dronc alone without simultaneous co-expression of Dark is sufficient to induce an ectopic phenotype. The incomplete expansion of the adult wing in response to Dronc-only mis-expression is a prominent example [47].

### Implications of K78 mono-ubiquitylation for non-catalytic functions of Dronc

We also examined the role of K78 ubiquitylation in a catalytically inactive (C318A) Dronc background. *da*>*Flag*-*Dronc*^*C318A*^ fails to rescue any of the *dronc* null mutant phenotypes examined such as lethality, apoptosis and male genitalia rotation, and also fails to induce AiP. However, surprisingly, the ubiquitylation-defective and catalytically inactive double mutant of Dronc (*da*>*Flag*-*Dronc*^*K78RC318A*^) does rescue the lethality and male genitalia rotation phenotypes of *dronc* null mutants and promotes AiP (Figs 4 and 5). The rescue of these phenotypes is not the result of restoring the catalytic activity of Dronc^K78RC318A^ by the K78R mutation because *in vitro* cleavage assays demonstrated that the effector caspase DrICE was not processed and *in vivo* IOC apoptosis was not rescued in *dronc* null mutants (Fig 3A and 3B; Fig 4), indicating that Dronc^K78RC318A^ has no catalytic and thus no apoptotic activity. Therefore, even though Dronc^K78R^ is released from inhibitory ubiquitylation, it still needs its catalytic activity to execute apoptosis. *Flag*-*Dronc*^*K78RC318A*^ is an intragenic suppressor of several, but not all, phenotypes associated with loss of the catalytic activity of Dronc. Therefore, the Flag-*Dronc*^*K78RC318A*^ transgene offers unique opportunities to identify and characterize apoptosis- (effector caspase-) independent functions of Dronc and to distinguish them from effector caspase-dependent ones.

These results allow making the following important conclusions about Dronc function. Firstly, the pupal lethality (which is actually a strong semi-lethality) associated with *dronc* null mutations is not only due to loss of the catalytic (enzymatic) activity. It appears that some non-catalytic functions of Dronc are also very important for survival of the animal. Loss of the catalytic activity may contribute to the pupal lethality, but it may not be the underlying cause. This conclusion may not apply to the embryonic lethality of *dronc* germline clones [48]. Secondly, because we demonstrated that K78 mono-ubiquitylation controls the interaction of Dronc with Dark, it appears that Dronc^K78RC318A^ executes its non-enzymatic functions also through increased interaction with Dark. Thus, increased interaction with Dark is sufficient for induction of several non-apoptotic functions of Dronc such as AiP. Thirdly, it is a hot debate in the caspase field how caspases are restrained from inducing apoptotic death during non-apoptotic processes [75–77]. However, our results imply that at least for the caspase Dronc, its catalytic activity is not strictly required for non-apoptotic processes, although it may contribute to it. Instead, it appears that K78 mono-ubiquitylation controls activation of Dronc for non-apoptotic processes without requiring the catalytic function of Dronc.

### Evolutionary considerations

Dronc is considered to be the *Drosophila* Caspase-9 ortholog; however it has more protein similarity to mammalian Caspase-2 [78]. Alignment of the CARD domains of Dronc and Caspase 2 showed that K78 is not a conserved residue. However, there are two conserved Lys residues at positions 20 and 65. It is possible that Caspase-2 may be ubiquitylated at one of these residues and this ubiquitylation may play a role in formation of the PIDDosome, an apoptosome-like protein complex required for Caspase-2 activation [79]. On the other hand, Caspase-9 does not have any Lys residue in its CARD domain. It is possible that the CARD domain of Caspase-9 has not evolved an ubiquitylation control mechanism because the interaction between Caspase-9 and Apaf-1 is not rate limiting for Caspase-9 activation (Cytochrome c release is). Nevertheless, similar to Dronc, mature Caspase-9 ubiquitylation has been shown *in vitro* [80], suggesting that Caspase-9 activation may be controlled by ubiquitylation after activation in the Apaf-1 apoptosome.

Our work highlights a mechanism where Dronc’s activity is negatively regulated through mono-ubiquitylation that interferes with its interaction partner Dark. This work may help understanding the similarities and differences of caspase activation in mammalian and *Drosophila* apoptosomes.

## Materials and methods

### Immunoprecipitations and immunoblotting

Embryos, 3^rd^ instar wandering larvae, 1–2 days old pupae and heads of adult flies were lysed in 100 ul of SDS lysis buffer containing 2% SDS, 150 nM NaCl, 10 mM TrisHCl, 20 uM NEM and protease inhibitors (Promega), respectively. The samples were sonicated for 10 seconds twice after they were boiled at 100°C for 10 minutes. 900 ul of dilution buffer (10 mM TrisHCl, 150 mM NaCl, 2 mM EDTA and 1% Triton-X) was added to the samples and samples were rotated at 4°C for 1 hour before centrifugation for 30 minutes. Protein concentrations of supernatants were measured by Bradford Assay. 30 ug and 425 ug of total protein were used for western blots and IPs, respectively. IP was performed with anti-Flag M2 magnetic beads (Sigma-Aldrich M8823) overnight at 4°C with rocking. 100 ul of 150 ng/ul Flag peptide in TBS was used for elution which took place at 4°C for 2 hours. 25 ul of eluted protein was used for western blotting. Dilutions of antibodies used are as follows: anti-Flag M2 antibody (1:1000), FK2 and FK1 (Enzo Life Sciences– 1:200), anti-Actin (Millipore Mab1501- 1:2000).

For ubiquitin pull-down assays, 3^rd^ instar larvae were collected and lysed in urea lysis buffer containing 8 M Urea, 0.1 M NaH_2_PO_4_, 0.01 M TrisHCl, 0.05% Tween 20, pH 8.0 and protease inhibitors. IP was performed with Nickel-NTA magnetic agarose beads (Qiagen 36111) at 4°C overnight with rocking. 60 ul of 250 mM of Imidazole in urea lysis buffer (pH 4.5) was used for elution. 30 ul of eluted protein was analyzed by western blot. Anti-His antibody (Thermo Scientific-Fisher MA1-21315) was used at 1:1000 dilution.

For co-IPs, 3^rd^ instar larvae were collected and lysed in NP40 buffer (20 mM TrisHCl pH 8.0, 137 mM NaCl, 1% NP40, 2 mM EDTA and protease inhibitors). IP was performed with GFP-Trap (ChromoTek) magnetic beads at 4°C overnight. GFP-Dark protein was eluted with 50 ul of 0.2M Glycine buffer pH 2.5. 25 ul of eluted protein was used for western blot. Anti-GFP antibody (Thermo Scientific-Fisher MA5-15256) was used at 1:200 for IP-western blots, 1:1000 for western blots.

Immunoblot band intensities are quantified with GelQuantNET software provided by biochemlabsolution.com. Uncropped immunoblots are presented in S7 and S8 Figs.

### Deubiquitylation assay

Immunoprecipitated Flag-Dronc was incubated with 3 ul of USP2 enzyme (Boston Biochem E-504) in deubiquitylation assay solution (50 mm EDTA, 100 mm DTT, 50 mm Tris-HCl and 150 mm NaCl) for 90 min at 37°C.

### LC-MS/MS analysis

Flag-Dronc was immunoprecipitated from larval and pupal *da*>*Flag-Dronc* extracts as described above. 1 mg of protein was used for IPs. Elutions of eight IPs were pooled and concentrated with 0.5 ml centrifugal tubes (Millipore UFC500324). *In vitro* ubiquitylation assays were performed as described previously [81]. Concentrated IP samples and *in vitro* ubiquitylated Dronc were loaded to 4–20% gradient SDS-PAGE gels. The gels were stained with Coomassie Blue Solution (Thermo Scientific-Fisher- 24590) and the 60 kDa band as well as higher molecular weight bands (for *in vivo* samples) were excised and submitted to MS Bioworks (Ann Arbor, MI). Samples were digested with Chymotrypsin and analyzed by LC-MS/MS. In the *in vitro* and *in vivo* samples, one peptide (K^78^ITQRGPTAY) carried the di-Glycine motif, characteristic for ubiquitylation.

### Fly work and generation of transgenic flies

The following fly stocks were used: *daughterless* (*da*)-*Gal4*; *GMR*-*Gal4*; *UAS*-*Flag*-*Dronc* [47]; *UAS*-*Flag*-*Dronc*^*wt*^; *UAS*-*Flag*-*Dronc*^*K78R*^, *UAS*-*Flag*-*Dronc*^*C318A*^ and *UAS*-*Flag*-*Dronc*^*K78RC318A*^ (this work); *UAS*-*6xHis*-*ubiquitin* (this work); *UAS*-*GFP*-*Dark* and *UAS*-*GFP*-*Dark*^*V*^ [17]; *dronc*^*I24*^ and *dronc*^*I29*^ [48]; *ey*>*p35* and *ey*>*hid*,*p35* [60]; *diap1*^*5*^ [26,27]. Please note that two *UAS*-*Flag*-*Dronc*^*wt*^ transgenes were used. The first one (a kind gift of Dr. Sally Kornbluth) was used in the initial phases of this work and has a random insertion on chromosome 3 [47]. The second one was obtained by phiC31 site-specific integration in the VK37 landing site on chromosome 2 (see below). This line was used in combination with *UAS*-*Flag*-*Dronc*^*K78R*^, *UAS*-*Flag*-*Dronc*^*C318A*^ and *UAS*-*Flag*-*Dronc*^*K78RC318A*^. All crosses were carried out at room temperature. 3L MARCM clones were induced by heat shocking L1 larvae at 37°C for 45 minutes as described [82]. Co-expression of *UAS-GFP-Dark* and *UAS-Flag-Dronc* transgenes was controlled by GAL80^ts^ [83]. Temperature shift was performed at 29°C for 24 h. 3^rd^ instar larvae were collected for lysis immediately after temperature shift.

Wild-type and mutant *UAS-Flag-Dronc* transgenic flies were generated by the phiC31 site-specific integration system [50,51]. Flag-Dronc-pTFW and Flag-Dronc^C318A^-pAFW vectors were kind gifts from Dr. Sally Kornbluth. Flag-Dronc and Flag-Dronc^C318A^ were cloned into pENTR3C vector. Point mutations were generated by site-directed mutagenesis. AttB site for site-specific integration was cloned into pTFW vector (DGRC—1115). Wild-type and mutant Flag-Dronc coding sequences were cloned into attB-pTFW vector by Gateway Cloning Technology (Gateway LR Clonase II Enzyme Mix). Plasmids were sent to Genetivision for injection. VK37 landing site was used for phiC31 integration [84].

*UAS-6xHis-Ubiquitin* transgenic flies were generated by random integration (Bestgene) of a pUAST-6xHis-Ubiquitin construct created by inserting a KpnI-XbaI fragment of N-terminal 6xHis human Ubiquitin pcDNA3.1 into pUAST [46]. Expression of *6xHis-Ubiquitin* was validated by FK2 Western blotting of urea-based lysis/Ni^2+^-based purification lysates generated from 20 adult *da*-*GAL4*;*UAS*-*6xHis*-Ubiquitin flies.

### Immunohistochemistry

3^rd^ instar larval brain lobes with eye discs were dissected in PBS and fixed in 4% PFA. Samples were blocked with 2% NDS in PBST and stained with c-Dcp-1 (Cell Signaling 9578–1:100) and anti-Flag (1:200) antibodies [85]. TUNEL was performed as described [86]. For pupal dissections, pupae were aged to 42 h-48 h APF. Pupal discs were dissected, fixed and stained for c-Dcp-1 and Dlg (DSHB 4F3 anti-disc large -1:100) [85]. Imaginal discs were mounted in Vectashield and imaged by confocal microscopy.

### Caspase activity assays

Caspase activity assays were performed as described [86,87]. Briefly, adult heads were lysed in caspase assay buffer (50 mM HEPES pH 7.5, 100 mM NaCl, 1 mM EDTA, 0.1% CHAPS, 10% sucrose, 5 mM DTT, 0.5% TritonX-100, 4% glycerol and protease inhibitors). Protein concentration was measured with Bradford Assay. 40 ug of protein was incubated with 100 uM of DEVD-AMC caspase substrate (MP Biomedicals 195868) in a final volume of 100 ul of caspase assay buffer. Fluorescence was measured with spectrophotometer (excitation 385 nM emission 460 nM) at 15 min intervals for 3 hours at 37°C. Each experiment was done at least three times.

### Caspase cleavage assay

For *in vitro* cleavage assays, wild type and mutant Dronc coding sequences were cloned into pET-28a plasmid to yield 6xHis fusion proteins. Generated plasmids were transformed to BL21(DE3)pLysS competent cells (Promega L1191). 50 ul of bacterial culture was grown at 37°C. Plasmid expression was induced by 0.2 mM IPTG for 3 h at 30°C as described [88]. Bacterial pellets were lysed with 4 ml of CellLytic B Cell Lysis Reagent (Sigma-Aldrich B7435) after adding 0.2 mg/ml Lysozyme, 50 units/ml Benzonase and 1X protease inhibitor (Roche).

Drice^C211A^-pET23b plasmid was a kind gift from Dr. Guy Salvesen [53]. Drice^C211A^ coding sequence was cloned into PT7CFE1-Nmyc plasmid (Thermo Scientific 88863). Myc-Drice^C211A^ protein was generated by using TNT Rabbit Reticulocyte Lysate System (Promega L4610). 4 ul of Myc-Drice^C211A^ protein was incubated with 100 ug of wild-type and mutant 6xHis-Dronc protein in caspase assay buffer (100 mM Hepes pH 7.5, 0.1% CHAPs, 10% sucrose, 10 mM DTT, 50 mM Nacl, 0.5 mM EDTA, protease inhibitor). The reaction was incubated at 30°C for 3 hours [54] and analyzed by western blotting. Anti-Myc antibody (Santa Cruz SC40) was used at 1:200 concentration.

### Statistical analyses

Student’s t-test is used in all graphical analyses with parametric statistics. Crosses are repeated at least three times. Numbers of fly eyes used for area calculation and staining intensity are indicated in corresponding figures. The quantification of eye size was done using the Histogram function in Photoshop.

## Supporting information

S1 Fig *Flag-Dronc*^*wt*^ is functional. (**A**) *Flag-Dronc* can rescue the lethality associated with *dronc* null mutations. (**B**) *Flag-Dronc* can be activated in the apoptosome. Expression of either *da*>*Flag-Dronc* or *GMR*-*Dark* does not lead to any caspase (cleaved caspase-3, cc3) activity. However, when these transgenes are co-expressed (*da>Flag-Dronc+GMR-Dark*), caspase activity is increased in the posterior domain.
(TIF)

S2 Fig LC-MS/MS analysis shows that Dronc is ubiquitylated at K78. **(A,B)** Of the peptides obtained by Chymotrypsin digests of immunoprecipated Dronc from larval and pupal extracts under surviving conditions (A), only the peptide K^78^ITQRGPT was found to carry the di-Glycine signature indicative of ubiquitin modification. di-Glycine is derived from conjugated ubiquitin and adds 114 Da to this peptide. Correspondingly, all b peaks of this peptide obtained under surviving conditions (A) are shifted compared to the b peaks under apoptotic conditions (B; see asterisk at peak b1 as example). **(C,D**) LC-MS/MS analyses of *in vitro* ubiquitylated Dronc with Diap1 as E3 ligase and either human UBE2D2 (C) or *Drosophila* UBCD1 (D) as E2 conjugating enzymes show that K78 can be ubiquitylated by DIAP1. Arrows indicate 114 Da mass shift due to ubiquitylation on K78.
(TIF)

S3 Fig Heterozygous *diap1*^*5*^ mutant strongly enhances *GMR>FlagDronc*^*wt*^ +*GFP-Dark* eye phenotype, but only weakly enhances *GMR>Flag-Dronc*^*K78R*^*+GFP-Dark*. **(A-C)** Loss of one copy of *diap1* strongly enhances eye phenotype of *GMR>Flag-Dronc*^*wt*^+*GFP-Dark* (quantified in B) and causes a significant increase in lethality (quantified in C). In contrast, *diap1* heterozygosity only weakly enhances *GMR>Flag-Dronc^K78R^+GFP-Dark* eye phenotype (quantified in B) and lethality (quantified in C). (**B**) Quantification of eye size phenotypes in (A). n = 9 for *GMR>Flag-Dronc*^*wt*^+*GFP-Dark*, n = 11 for *GMR>Flag-Dronc*^*wt*^+*GFP-Dark+diap1*^*5*^, n = 8 for *GMR>Flag-Dronc*^*K78R*^+*GFP-Dark*, *n = 11 for GMR>Flag-Dronc*^*K78R*^+*GFP-Dark+diap1*^*5*^. **(C)** Quantification of eclosion rates of *GMR>Flag-Dronc*^*wt*^+*GFP-Dark and GMR>Flag-Dronc*^*K78R*^+*GFP-Dark* with or without loss of one copy of *diap1*. For quantifications, the student’s t-test was used. Error bars are SD. * P<0.05; *** P<0,001; ns—not significant.
(TIF)

S4 Fig Cleavage resistant GFP-Dark^V^ can form a more functional apoptosome with Flag-Dronc^K78R^ than with Flag-Dronc^wt^. **(A)** Expression of *GMR*>*Flag*-*Dronc*^*K78R*^+*GFP*-*Dark* resulted in significantly smaller eyes than *GMR*>*Flag*-*Dronc*^*wt*^ +*GFP*-*Dark*. Expression of *GMR>GFP-Dark*^*V*^ alone does not have any eye phenotype. (**B**) Quantification of eye size phenotypes in (A). n = 10 for each genotype. **(C)** Eclosion rates of flies expressing *GMR*>*Flag*-*Dronc*^*K78R*^ +*GFP*-*Dark*^*V*^ are significantly smaller than *GMR*>*Flag*-*Dronc*^*wt*^ +*GFP*-*Dark*^*V*^. For quantifications, the student’s t-test was used. Error bars are SD. ** P<0.01.
(TIF)

S5 Fig Both *Flag-Dronc*^*K78RC318A*^ and *Flag-Dronc*^*C318A*^ cannot rescue the wing phenotype of *dronc* null mutants. Compared to control flies (A, *w*^*1118*^), wings from *dronc* null mutants are held-out, often irregularly shaped and less transparent (B). Often one wing is missing (see (F)). *da*>*Flag*-*Dronc*^*K78RC318A*^ (E) and *da*>*Flag*-*Dronc*^*C318A*^ (F) do not rescue this phenotype. In contrast, Flag-Dronc^wt^ and Flag-Dronc^K78R^ rescue the wing phenotype of *dronc* null mutants (C,D). However, these wings are not fully expanded due to ectopic apoptosis of Bursicon-expressing neurons (for details see reference [47]). This observation suggests that there are conditions where mis-expression of only Dronc is sufficient to induce apoptosis without simultaneous expression of Dark, presumably because of endogenous Dark levels are high enough.
(TIF)

S6 Fig *Flag-Dronc*^*K78R*^ and *Flag-Dronc*^*K78RC318A*^ can induce a head capsule overgrowth phenotype. **(A)** Expression of *Flag-Dronc*^*wt*^, *Flag-Dronc*^*K78R*^ and *Flag-Dronc*^*K78RC318A*^ in *ey*>*p35* background can induce overgrowth phenotypes. Overgrowth is characterized by expanded head cuticle with pattern duplications such as bristles and ocelli. In contrast, *Flag-Dronc*^*C318A*^ cannot induce this phenotype. **(B)** Expression of indicated *Flag-Dronc* constructs with *ey-GAL4* does not lead to any eye phenotype. For quantifications, the student’s t-test was used. Error bars are SD. * P<0.05; ** P<0.01; ns—not significant.
(TIF)

S7 Fig Uncropped immunoblots of Fig 1. (TIF)

S8 Fig Uncropped immunoblots of Figs 3 and 4. (TIF)
