# Supplementary figures and images for: An inhibitory mono-ubiquitylation of the Drosophila initiator caspase Dronc functions in both apoptotic and non-apoptotic pathways
Source: PLoS Genet. 2017 Feb 16;13(2):e1006438. doi: 10.1371/journal.pgen.1006438 (PMC5313150; doi:10.1371/journal.pgen.1006438)

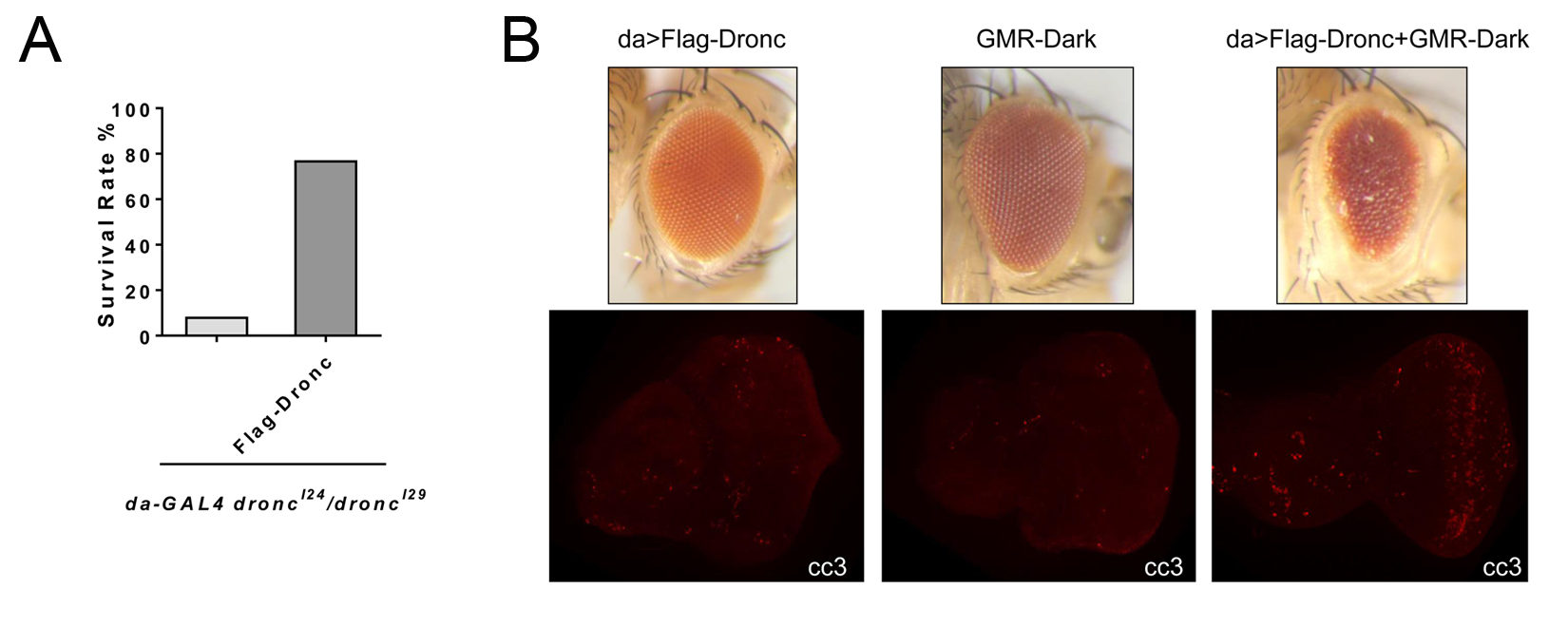

Supplement: S1 Fig — (A) Flag-Dronc can rescue the lethality associated with dronc null mutations. (B) Flag-Dronc can be activated in the apoptosome. Expression of either da>Flag-Dronc or GMR-Dark does not lead to any caspase (cleaved caspase-3, cc3) activity. However, when these transgenes are co-expressed (da>Flag-Dronc+GMR-Dark), caspase activity is increased in the posterior domain. (TIF) [file pgen.1006438.s001.tif]

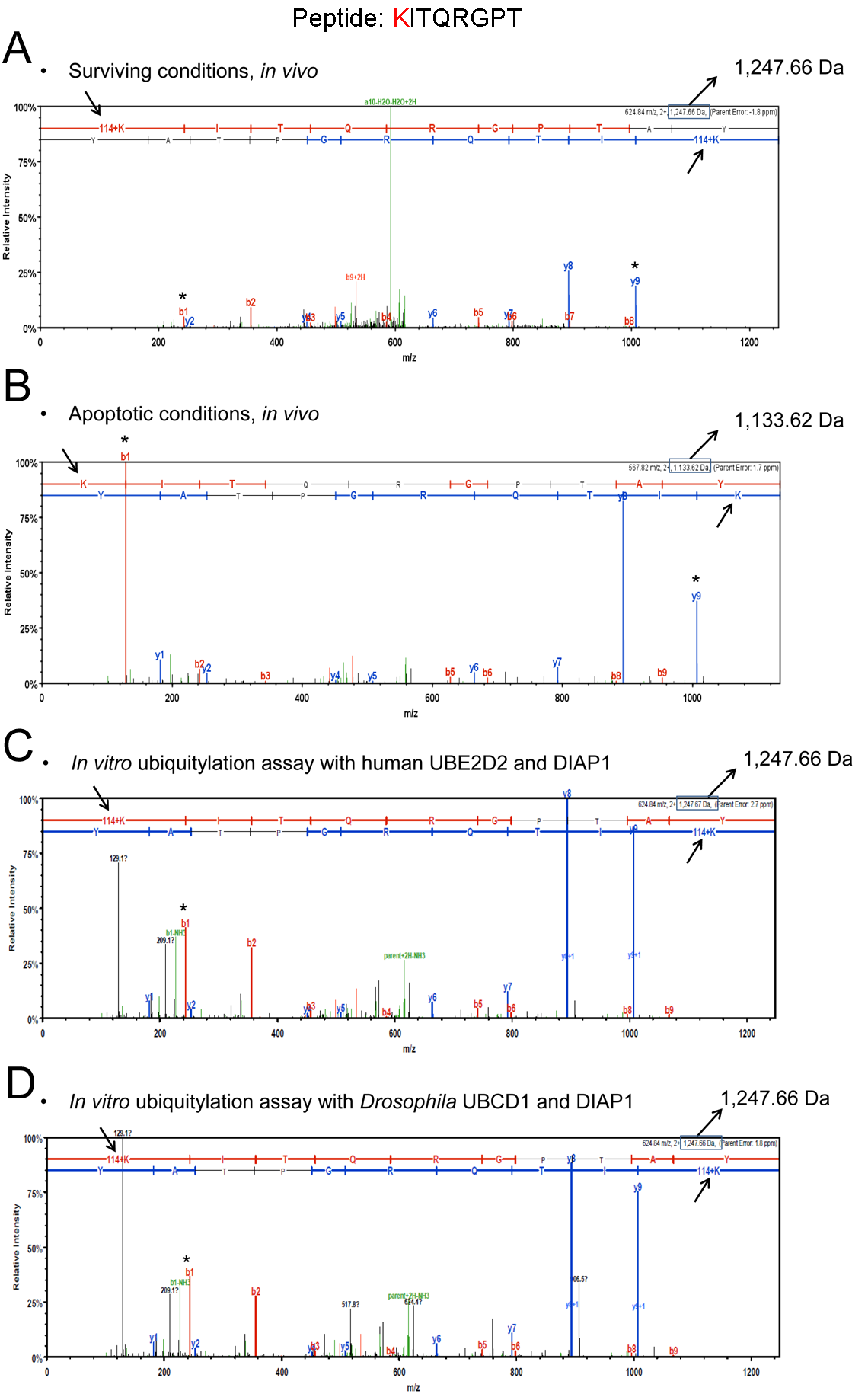

Supplement: S2 Fig — (A,B) Of the peptides obtained by Chymotrypsin digests of immunoprecipated Dronc from larval and pupal extracts under surviving conditions (A), only the peptide K78ITQRGPT was found to carry the di-Glycine signature indicative of ubiquitin modification. di-Glycine is derived from conjugated ubiquitin and adds 114 Da to this peptide. Correspondingly, all b peaks of this peptide obtained under surviving conditions (A) are shifted compared to the b peaks under apoptotic conditions (B; see asterisk at peak b1 as example). (C,D) LC-MS/MS analyses of in vitro ubiquitylated Dronc with Diap1 as E3 ligase and either human UBE2D2 (C) or Drosophila UBCD1 (D) as E2 conjugating enzymes show that K78 can be ubiquitylated by DIAP1. Arrows indicate 114 Da mass shift due to ubiquitylation on K78. (TIF) [file pgen.1006438.s002.tif]

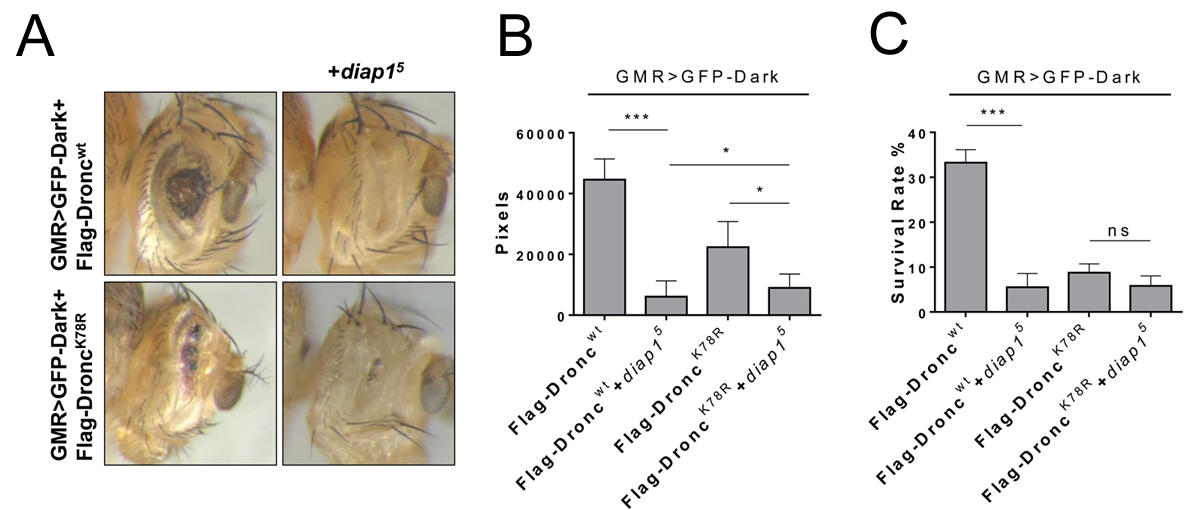

Supplement: S3 Fig — (A-C) Loss of one copy of diap1 strongly enhances eye phenotype of GMR>Flag-Droncwt+GFP-Dark (quantified in B) and causes a significant increase in lethality (quantified in C). In contrast, diap1 heterozygosity only weakly enhances GMR>Flag-DroncK78R+GFP-Dark eye phenotype (quantified in B) and lethality (quantified in C). (B) Quantification of eye size phenotypes in (A). n = 9 for GMR>Flag-Droncwt+GFP-Dark, n = 11 for GMR>Flag-Droncwt+GFP-Dark+diap15, n = 8 for GMR>Flag-DroncK78R+GFP-Dark, n = 11 for GMR>Flag-DroncK78R+GFP-Dark+diap15. (C) Quantification of eclosion rates of GMR>Flag-Droncwt+GFP-Dark and GMR>Flag-DroncK78R+GFP-Dark with or without loss of one copy of diap1. For quantifications, the student’s t-test was used. Error bars are SD. * P<0.05; *** P<0,001; ns—not significant. (TIF) [file pgen.1006438.s003.tif]

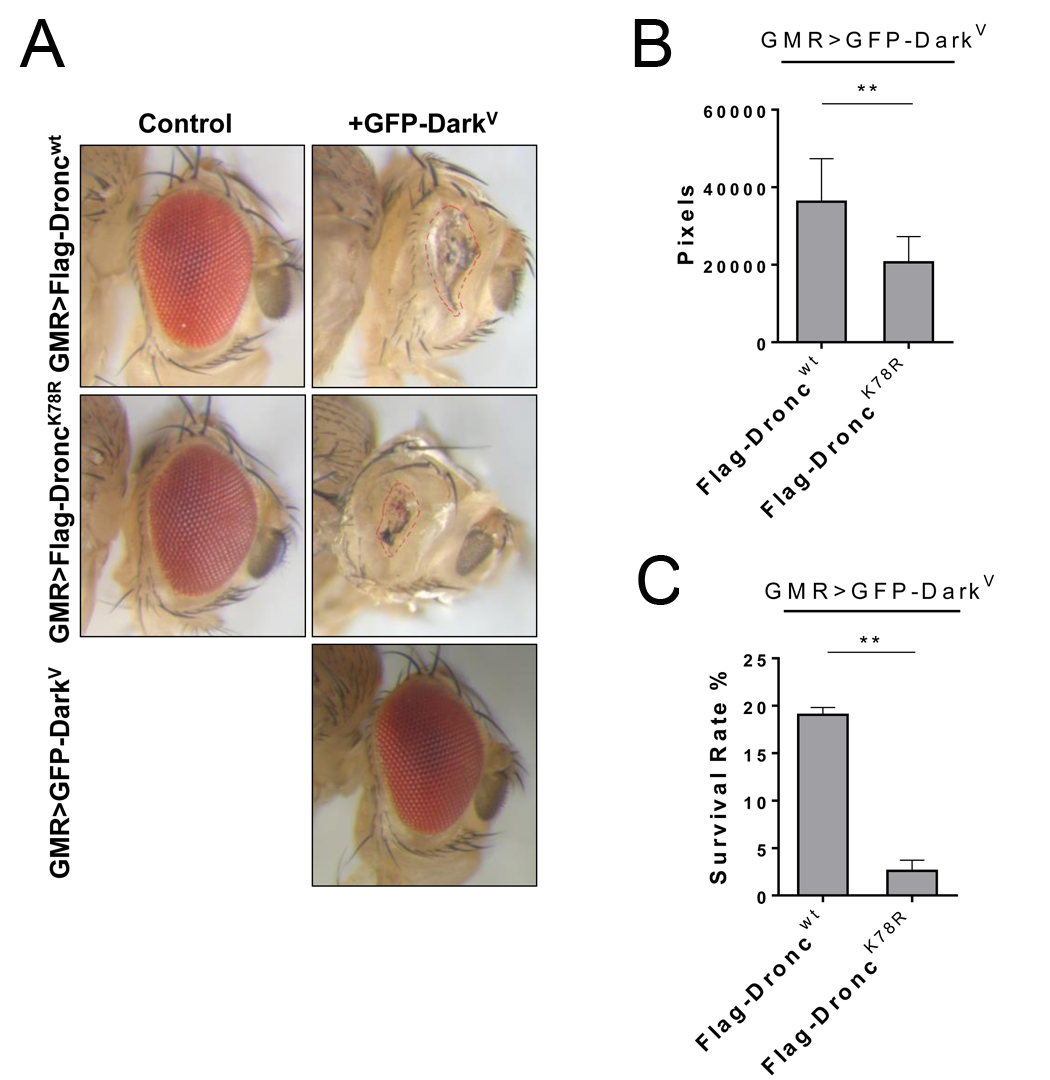

Supplement: S4 Fig — (A) Expression of GMR>Flag-DroncK78R+GFP-Dark resulted in significantly smaller eyes than GMR>Flag-Droncwt +GFP-Dark. Expression of GMR>GFP-DarkV alone does not have any eye phenotype. (B) Quantification of eye size phenotypes in (A). n = 10 for each genotype. (C) Eclosion rates of flies expressing GMR>Flag-DroncK78R +GFP-DarkV are significantly smaller than GMR>Flag-Droncwt +GFP-DarkV. For quantifications, the student’s t-test was used. Error bars are SD. ** P<0.01. (TIF) [file pgen.1006438.s004.tif]

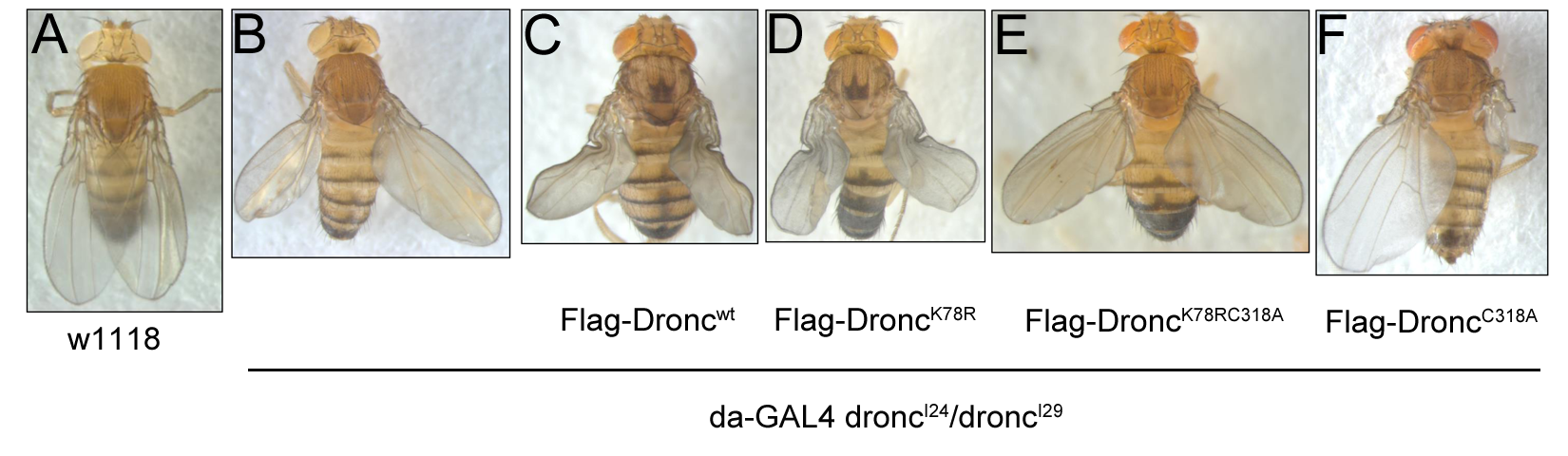

Supplement: S5 Fig — Compared to control flies (A, w1118), wings from dronc null mutants are held-out, often irregularly shaped and less transparent (B). Often one wing is missing (see (F)). da>Flag-DroncK78RC318A (E) and da>Flag-DroncC318A (F) do not rescue this phenotype. In contrast, Flag-Droncwt and Flag-DroncK78R rescue the wing phenotype of dronc null mutants (C,D). However, these wings are not fully expanded due to ectopic apoptosis of Bursicon-expressing neurons (for details see reference [47]). This observation suggests that there are conditions where mis-expression of only Dronc is sufficient to induce apoptosis without simultaneous expression of Dark, presumably because of endogenous Dark levels are high enough. (TIF) [file pgen.1006438.s005.tif]

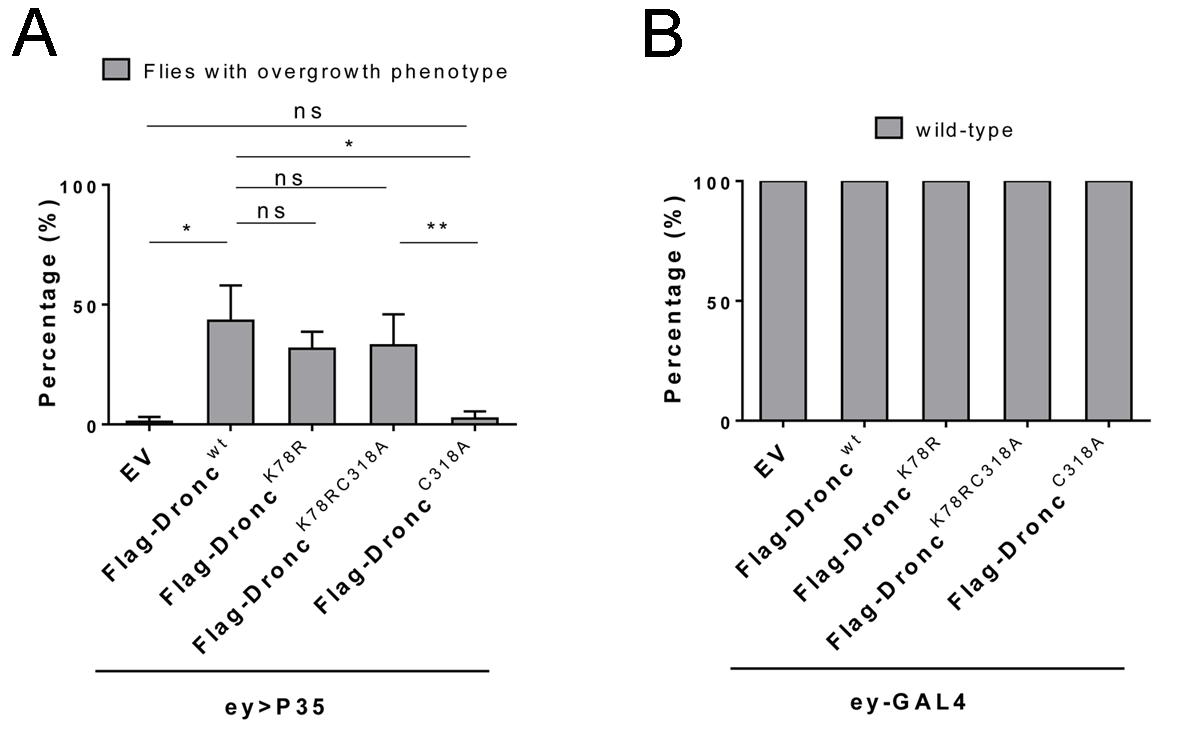

Supplement: S6 Fig — (A) Expression of Flag-Droncwt, Flag-DroncK78R and Flag-DroncK78RC318A in ey>p35 background can induce overgrowth phenotypes. Overgrowth is characterized by expanded head cuticle with pattern duplications such as bristles and ocelli. In contrast, Flag-DroncC318A cannot induce this phenotype. (B) Expression of indicated Flag-Dronc constructs with ey-GAL4 does not lead to any eye phenotype. For quantifications, the student’s t-test was used. Error bars are SD. * P<0.05; ** P<0.01; ns—not significant. (TIF) [file pgen.1006438.s006.tif]

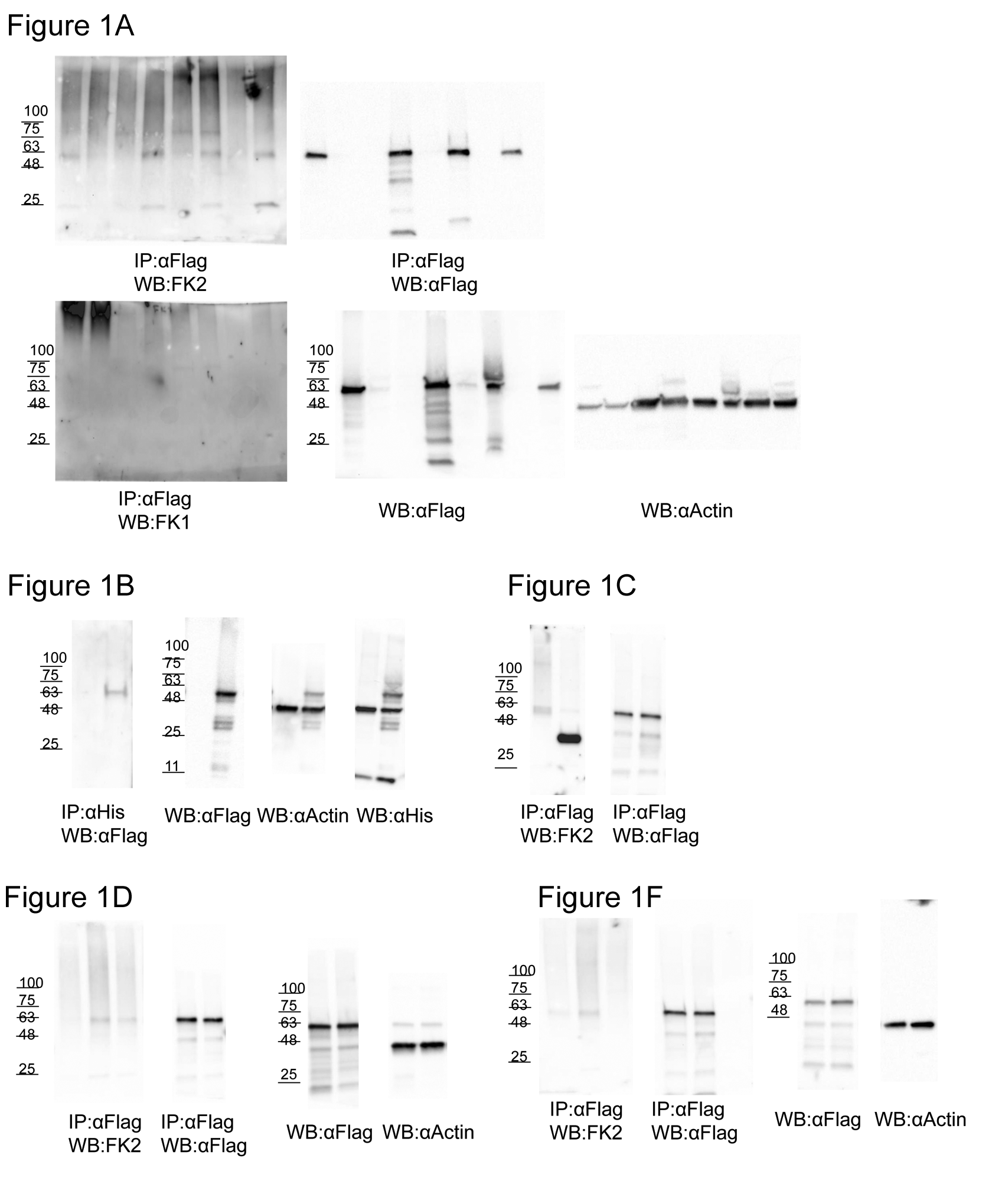

Supplement: S7 Fig — (TIF) [file pgen.1006438.s007.tif]

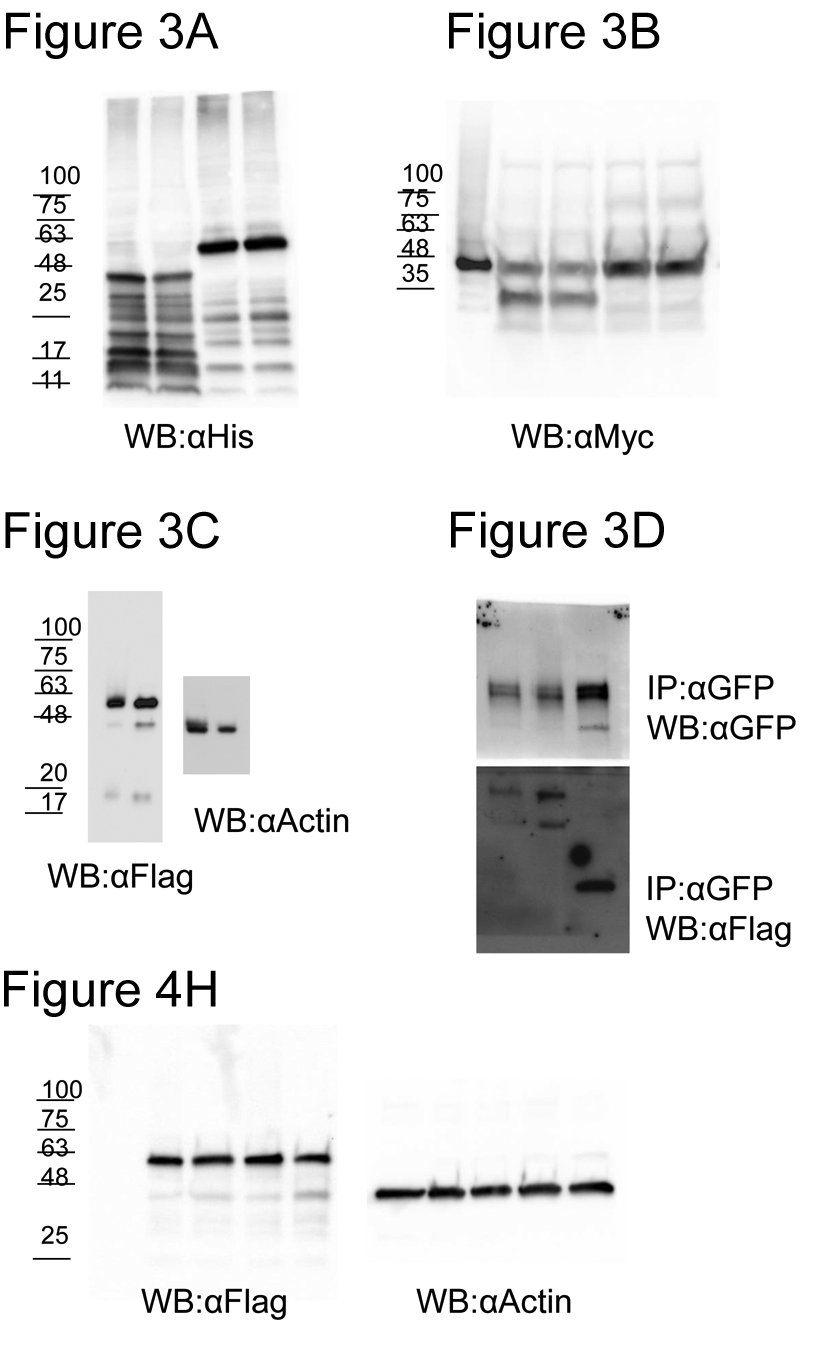

Supplement: S8 Fig — (TIF) [file pgen.1006438.s008.tif]
